# Urinary metabolomics may improve prediction of overall survival beyond tumor stage in colorectal cancer: results from the ColoCare study

**DOI:** 10.1007/s11306-026-02516-3

**Published:** 2026-09-11

**Authors:** Tengda Lin, Boyi Guo, Victoria M. Bandera, David B. Liesenfeld, Jincheng Shen, Benjamin Haaland, Paul A. Stewart, Kenneth M. Boucher, Patricia A. Erickson, Sheetal Hardikar, Victoria Damerell, Doratha A. Byrd, Jane C. Figueiredo, Adetunji T. Toriola, David Shibata, Erin M. Siegel, Christopher I. Li, Alexis B. Ulrich, Christoph Kahlert, Daniel O. Scharfstein, Biljana Gigic, Cornelia M. Ulrich, Jennifer Ose

**Affiliations:** 1 Huntsman Cancer Institute, University of Utah, Salt Lake City, UT USA; 2 Department of Population Health Sciences, University of Utah, Salt Lake City, UT USA; 3 Division of Preventive Oncology, National Center for Tumor Diseases (NCT), German Cancer Research Center (DKFZ), Heidelberg, Germany; 4 Department of Internal Medicine, University of Utah, Salt Lake City, UT USA; 5 Department of Nutrition and Integrative Physiology, University of Utah, Salt Lake City, UT USA; 6 Department of General, Visceral, and Transplantation Surgery, Heidelberg University Hospital, Heidelberg, Germany; 7 H. Lee Moffitt Cancer Center and Research Institute, Tampa, FL USA; 8 Department of Medicine, Cedars-Sinai Cancer, Cedars-Sinai Health Sciences University, Los Angeles, CA USA; 9 Washington University School of Medicine in St. Louis, St. Louis, MO USA; 10 Department of Surgery, University of Tennessee Health Science Center, Memphis, TN USA; 11 Public Health Sciences Division, Fred Hutchinson Cancer Center, Seattle, WA USA; 12 Lukaskrankenhaus Neuss, Neuss, Germany; 13 Department of Information and Communication, Faculty for Media, Information and Design, University of Applied Sciences and Arts, Hannover, Germany

**Keywords:** Urinary metabolomics, Overall survival prediction, Penalized Cox models

## Abstract

**Background:**

Colorectal cancer (CRC) is a leading cause of cancer-related mortality. Prognosis is primarily guided by tumor stage despite substantial molecular heterogeneity. Urinary metabolomics may capture systemic and tumor-related biology beyond staging and could improve prognostic assessment. We hypothesized that incorporating urinary metabolomic profiles would improve overall survival (OS) prediction performance compared with a stage- and age-based reference model.

**Method:**

A total of n = 76 stage I-IV CRC patients recruited as part of the ColoCare Study in Heidelberg Germany with pre-surgery urinary metabolomics were included (23 deaths; median follow-up 3.03 years). Four metabolomics-based penalized Cox models adjusted for tumor stage and age at diagnosis were developed using LASSO, adaptive LASSO, spike-and-slab LASSO, and iterative sure independence screening (iSIS)-LASSO. Model discrimination was assessed using Harrell’s C-index and time-dependent AUC based on the nested cross-validation.

**Results:**

Compared with the reference model (Cox model including only tumor stage and age at diagnosis), all metabolomics-based models provided better discrimination. The spike-and-slab LASSO Cox model demonstrated the best performance, achieving a C-index of 0.75 (*vs.* 0.68) and consistently higher time-dependent AUCs at 1–5 years of follow-up, with a peak AUC of 0.76 at year 3 (*vs.* 0.68). Three urinary metabolites were consistently selected across all metabolomics-based models: indolelactate, 2-hydroxyisobutyrate and a uridine-like metabolite.

**Conclusions:**

Urinary metabolomics may improve CRC OS prediction beyond tumor stage and age at diagnosis, especially with the spike-and-slab LASSO Cox model. These results support urinary metabolomics as a promising noninvasive prognostic tool that merits external validation.

**Supplementary Information:**

The online version contains supplementary material available at https://doi.org/10.1007/s11306-026-02516-3.

## Introduction

Colorectal cancer (CRC) is the third most common type of cancer worldwide (World Health Organization, 2024), with over 1.9 million annual cases and 0.9 million deaths per year reported in 2022 (International Agency for Research on Cancer, 2024). In the US, CRC is the second leading cause of cancer death, with a 5-year relative survival rate of 65% (Siegel et al., 2026a, 2026b). Prognosis of CRC is primarily based on tumor stage at diagnosis, despite the heterogeneity of molecular characteristics and treatment response (Koncina et al., 2020; Lawler et al., 2018; Rosati et al., 2022). Consequently, relying primarily on tumor stage for prognostic assessment may not adequately support individualized risk stratification and can lead to over-treatment, with associated patient and economic burdens (Kneuertz et al., 2015; Scheele et al., 2018). Additional biomarkers that capture the functional consequences of molecular heterogeneity beyond conventional clinical factors may improve prognostic assessment.

Metabolomics is well suited to address this need because it captures the downstream products of cellular regulatory processes and therefore reflects the integrated functional state of both the tumor and the host (Fiehn, 2002; Griffin & Shockcor, 2004). Metabolic dysregulation is a hallmark of cancer (Schmidt et al., 2021), suggesting that metabolomic profiles may capture prognostically relevant biological variation beyond conventional clinical factors. However, prognostic application in CRC remains limited (Erben et al., 2018; Gold et al., 2022; Zhang et al., 2017). Urine-based metabolomics is attractive because its noninvasive collection and minimal processing offer accessible patient prognostic stratification (Bouatra et al., 2013; Brezmes et al., 2022). Urinary CRC biomarkers have been identified, including volatile organic compounds (Widlak et al., 2018), modified cytosine nucleosides (Guo et al., 2018), and polyamines (Nakajima et al., 2018; Venäläinen et al., 2018). Although primarily investigated for diagnosis, these biomarkers reflect metabolic alterations and suggest prognostic potential.

In CRC metabolomics, prognostic studies commonly use one of two strategies: (i) univariate screening, where metabolites are tested individually with multiple-testing correction and the significant metabolites are used to build survival models; or (ii) multivariate dimension reduction, typically partial least squares (PLS)–based, where latent components are derived, their corresponding component scores are used as predictors in Cox models to generate risk scores, and patients are stratified for Kaplan–Meier analysis (Bertini et al., 2012; Farshidfar et al., 2016; Jiménez et al., 2013; Liesenfeld et al., 2015; Ose et al., 2023; Phua et al., 2014; Qiu et al., 2014; Zaimenko et al., 2019). One major drawback of the univariate approach for feature selection is that it considers only marginal associations of individual features. Consequently, features with strong joint associations but weak marginal associations may be excluded (Fan & Lv, 2008). The PLS and orthogonal PLS (OPLS) methods are favored in chemometrics and adopted by metabolomics for dimension reduction (Trygg et al., 2007; Trygg & Wold, 2002; Wold, 1975). However, standard PLS uses all predictors to form latent components and does not yield sparse solutions, so all metabolites typically have nonzero loadings, complicating the interpretation of individual metabolite contributions. Although OPLS improves interpretability by separating predictive and orthogonal variation and has been extended into several variants (Magnanensi et al., 2021; Mehmood et al., 2012, 2020; Pinto, 2017), they still lack explicit variable selection. These limitations have led to increasing use of penalized Cox models for identifying prognostic metabolite signatures (Seum et al., 2025; Xu et al., 2022). However, penalized methods fit as a single model can be prone to model-selection instability. Because metabolites are highly correlated and true signals are likely sparse, the selected metabolite set can vary substantially with tuning parameters, random resampling/splits, or across datasets, limiting the selection stability and potentially hindering reproducibility and external validation in independent cohorts. (Jardillier et al., 2022; Waldmann et al., 2013).

To address these limitations, we applied a multi-model framework using four complementary high-dimensional variable-selection methods: least absolute shrinkage and selection operator (LASSO) (Simon et al., 2011; Tibshirani, 1996), adaptive LASSO (Zou, 2006), spike-and-slab LASSO (Rocková & George, 2014; Tang et al., 2017; Yi et al., 2019), and iterative sure independence screening (iSIS)-LASSO (Fan & Lv, 2008; Fan et al., 2009, 2010), to identify robust prognostic metabolite signatures from a prospective CRC cohort. By adjusting all models for key clinical covariates (tumor stage and age at diagnosis), we aim to identify prognostic information contributed by metabolomics beyond these established risk factors.

## Materials and methods

### Study population

We included patients recruited as part of the ColoCare Study in Heidelberg, Germany with available pre-surgery urinary metabolomics data. ColoCare is an international prospective cohort study designed to discover and validate novel prognostic biomarkers and risk prediction models (Ulrich et al., 2019). The study has been approved by the ethics committee of the University of Heidelberg and all study participants provided written informed consent. ColoCare participants were enrolled at the time of CRC diagnosis and followed longitudinally through in-person follow-up, systematic medical record review, and linkage to cancer registry and vital status records. Standardized medical record abstraction obtained detailed information on surgical procedures, oncologic treatments, and clinical outcomes. Vital status was ascertained through multiple sources, including local and external medical records, routine follow-up mailings, and linkage to state and national cancer and death registries.

### Biospecimen collection and assays

The details regarding the sample collection/preparation and data acquisition/pre-processing were described by Liesenfeld et al. (2015). Briefly, first-morning urine samples (predominantly fasting) were collected prior to surgery, aliquoted immediately, and stored at $-80\,^{\circ }\textrm{C}$. Patients reporting alcohol or tobacco use 24 h before urine collection were excluded. Sample processing followed Cheng et al. (2012), without urease treatment. Pooled quality control (QC) samples and Kovat’s alkane mixtures were injected with each analytical batch. Gas chromatography–mass spectrometry (GC-MS) analysis was performed on an Agilent 6890 GC/5973 MS single quadrupole system. Raw data were converted to netCDF format and processed with MZMine 2.0 (Pluskal et al., 2010). Chromatographic peaks were annotated at four levels (Sumner et al., 2007): Level 1 (reference standard available), Level 2 (electron impact (EI) spectral match (*>75%*) and Kovat’s retention indices (RI) match (*± 20*) to the National Institute of Standards and Technology (NIST) database), Level 3 (class-level EI spectral match *>75%*), Level 4 (unknown; excluded). We excluded metabolites with *>30%* coefficient of variance (CV) in QC samples (Dunn et al., 2011), and subsequently removed those with *>40%* missing values. Remaining missing values were imputed using the minimum observed value for each metabolite, consistent with our prior work showing no substantial differences between imputation methods (Liesenfeld et al., 2015). A total of 121 Level 1–3 annotated metabolites were included and normalized by sum (Sun & Xia, 2024), $log_{10}$-transformed, and scaled (mean-centered and divided by the standard deviation of each variable) for subsequent statistical analysis.

### Statistical method

####  Methods of modeling

The primary outcome was overall survival (OS), defined as the time from the date of CRC primary surgery to death from any cause or censoring at last follow-up. OS was modeled following the Cox proportional hazards framework, with the baseline hazard function estimated non-parametrically via the Breslow estimator (Breslow, 1975). We fit five Cox proportional hazard models: a clinical reference model including tumor stage (I–III *vs.* IV) and age at diagnosis (continuous), and four penalized models to handle the high-dimensional metabolite data, including LASSO, adaptive LASSO, spike-and-slab LASSO, iSIS-LASSO. LASSO imposes an $\ell _1$ penalty to produce sparse solutions and allows simultaneous estimation in settings with many potentially correlated predictors (Tibshirani, 1996). Adaptive LASSO uses data-driven penalty weights, and under suitable conditions enjoys “oracle” properties (Fan & Li, 2001), achieving consistent variable selection as if the true underlying model were known (Zou, 2006). Spike-and-slab LASSO adopts a Bayesian mixture (spike-and-slab) prior that better separates signal from noise and mitigates the shrinkage bias of standard LASSO (Tang et al., 2017; Yi et al., 2019). SIS was designed for ultrahigh-dimensional problems and can reduce dimensionality from very large *p* to a manageable subset while retaining the true signals with high probability (“sure screening” property) (Fan & Lv, 2008; Fan et al., 2009, 2010). In the iSIS–LASSO model, SIS is iteratively combined with LASSO, repeatedly screening and penalizing predictors until a stable set of signals is identified, thereby improving recovery of relevant metabolites. In all penalized models, tumor stage and age at diagnosis were included as unpenalized covariates. Detailed model specifications and tuning are provided in Supplement Table S1.1.

#### Prediction performance assessment

Model discrimination was assessed using the concordance index (C-index) (Harrell et al., 1996). The C-index measures the probability that, for a randomly selected pair of subjects whose survival times can be definitively ordered, the one who experiences the event earlier is assigned a higher predicted risk (shorter predicted survival); To assess how discrimination may change at different time points ($1^{st}, 2^{nd}, 3^{rd}, 4^{th}, \text {and}\ 5^{th}$ year after surgery), we evaluated the time-dependent area under the receiver operating characteristic curve (tAUC) (Heagerty et al., 2000), using the cumulative/dynamic (C/D) definition (Heagerty & Zheng, 2005; Kamarudin et al., 2017). We also calculated the integrated AUC (iAUC), defined as the weighted average of the tAUC over the follow-up period, to summarize overall discrimination across time. Both tAUC and iAUC were estimated using the method of Uno et al. (2007), which applies inverse probability of censoring weighting and yields consistent estimates under right censoring, assuming that censoring is independent of event times.

The above performance metrics were estimated using nested cross-validation, consisting of an outer loop (cross-validation layer) for performance estimation and an inner loop for hyperparameter tuning. In the outer loop, the data were randomly partitioned into five approximately equal-sized folds (outer folds) with similar event rates. Each outer fold served once as the test set, while the remaining four outer folds formed the training set. Within each outer training set, an inner 10-fold cross-validation was performed to select hyperparameters as needed. The model was then refit on the full outer training set using the selected hyperparameters and evaluated on the corresponding outer test set. Predictions from each outer test set were used to compute the C-index, tAUC, and iAUC; the performance was summarized by averaging each metric across the five outer test folds. This procedure prevents optimistic bias by ensuring that test data are never used for model tuning (Cawley & Talbot, 2010; Varma & Simon, 2006) and provides an approximately unbiased estimate of the generalization performance of the entire modeling procedure. The reported discrimination metrics reflect expected model performance on new data from the same underlying population (Stone, 1974; Hastie et al., 2009). To quantify uncertainty in discrimination performance for each modeling strategy, we performed a bootstrap analysis with 1000 replicates. For each bootstrap replicate, the complete nested cross-validation procedure, including hyperparameter tuning, feature selection, model fitting, and performance evaluation, was repeated. The resulting bootstrap distributions were used to derive 95% confidence intervals (CIs) for the C-index, iAUC, and tAUC using the empirical 2.5th and 97.5th percentiles (Efron & Tibshirani, 1993; Hastie et al., 2009). Within each bootstrap replicate, we also quantified the incremental discrimination attributable to metabolomics by calculating the difference in outer fold-averaged performance between each metabolomics-based model and the reference model. The resulting bootstrap distributions of these performance differences were then used to derive 95% CIs for the incremental discrimination.

#### Final model fit and calibration assessment

Final models were trained on all study participants, with hyperparameters selected *via* 10-fold cross-validation using the same criterion as in the nested procedure. As no independent test set was available, the performance of these final models cannot be directly evaluated; instead, nested cross-validation provides an approximately unbiased estimate of generalization performance of the modeling procedure. Selected predictors, corresponding effect estimates, and model calibration analyses are based on the final models.

Calibration was assessed by comparing observed and model-predicted event rates across groups defined by the model’s risk score (e.g., quartiles of the linear predictor) (Harrell et al., 1996; Royston & Altman, 2013). Within each group, observed event rates were calculated as event proportions and predicted event rates as the mean model-based event probability. Overall calibration was evaluated using a Poisson regression model, with a likelihood ratio test assessing whether observed event counts were consistent with model-expected counts across groups, thereby reflecting agreement between observed and predicted event rates (Berry, 1983; Atkinson et al., 2008; Crowson et al., 2016). Additional details are provided in Supplement Table S.1.2.

#### Sensitivity analysis

Given accumulating evidence that tumor site may be associated with OS in CRC (Liesenfeld et al., 2015; Petrelli et al., 2017; Shida et al., 2020; Ose et al., 2022), we conducted sensitivity analyses with additional adjustment for tumor site (rectum *vs.* colon). Tumor site was added to the clinical reference model and treated as an unpenalized covariate as other clinical variables in all penalized Cox models. All other model specification, tuning, and validation were unchanged. Results from the sensitivity analyses are presented in Supplement 2.

#### Simulation study

To evaluate the robustness and finite-sample properties of the penalized Cox models under the empirical correlation structure of the metabolomics data, we conducted a real data-based simulation study leveraging the empirical metabolite design matrix. We evaluated eighteen scenarios by systematically varying signal strength (three levels), multicollinearity (two levels), and censoring rates (three levels). To ensure stable performance estimates, we performed 200 iterations per scenario. Model performance was characterized by discrimination, true signal recovery, and coefficient estimation accuracy (see Supplement 3).

All statistical analyses were conducted in R (v4.4.2). Penalized Cox proportional hazards models were implemented using the glmnet package for LASSO and adaptive LASSO. Spike-and-slab LASSO models were developed using custom wrappers interfacing with the BhGLM package, and the iSIS-LASSO procedure was implemented using custom functions with glmnet utilities. Predictive performance evaluation regarding C-index was implemented *via* the survival package, while iAUC and tAUC were calculated using the survAUC package.

### Metabolite set enrichment analysis

For the metabolites identified from each model, we conducted metabolite set enrichment analysis using the MetaboAnalyst 6.0 (Pang et al., 2024). For the input reference set, we chose the ’Disease-associated metabolite sets’ (reported in urine) which contain 384 entries (accessed December 2025). The hypergeometric test was applied to evaluate whether a particular metabolite set is represented more than expected by chance within the given eference set. Benjamini-Hochberg procedure was used to adjusted p-values for multiple comparisons (Benjamini & Hochberg, 1995). The results are presented in Supplement Figures S1.1 and S1.2.

## Results

### Patient characteristics and survival

Seventy-six patients were included in the current study, of whom 23 (30%) died during follow-up. Median follow-up time was 3.03 years. The estimated survival probabilities were 0.93 (95% CI 0.88–0.99) at 6 months, 0.84 (95% CI 0.76–0.93) at 1 year, 0.80 (95% CI 0.71–0.89) at 2 years, and 0.59 (95% CI 0.45–0.79) at 5 years. Baseline patient characteristics are summarized in Table 1.

### Model discrimination

**Fig. 1:**
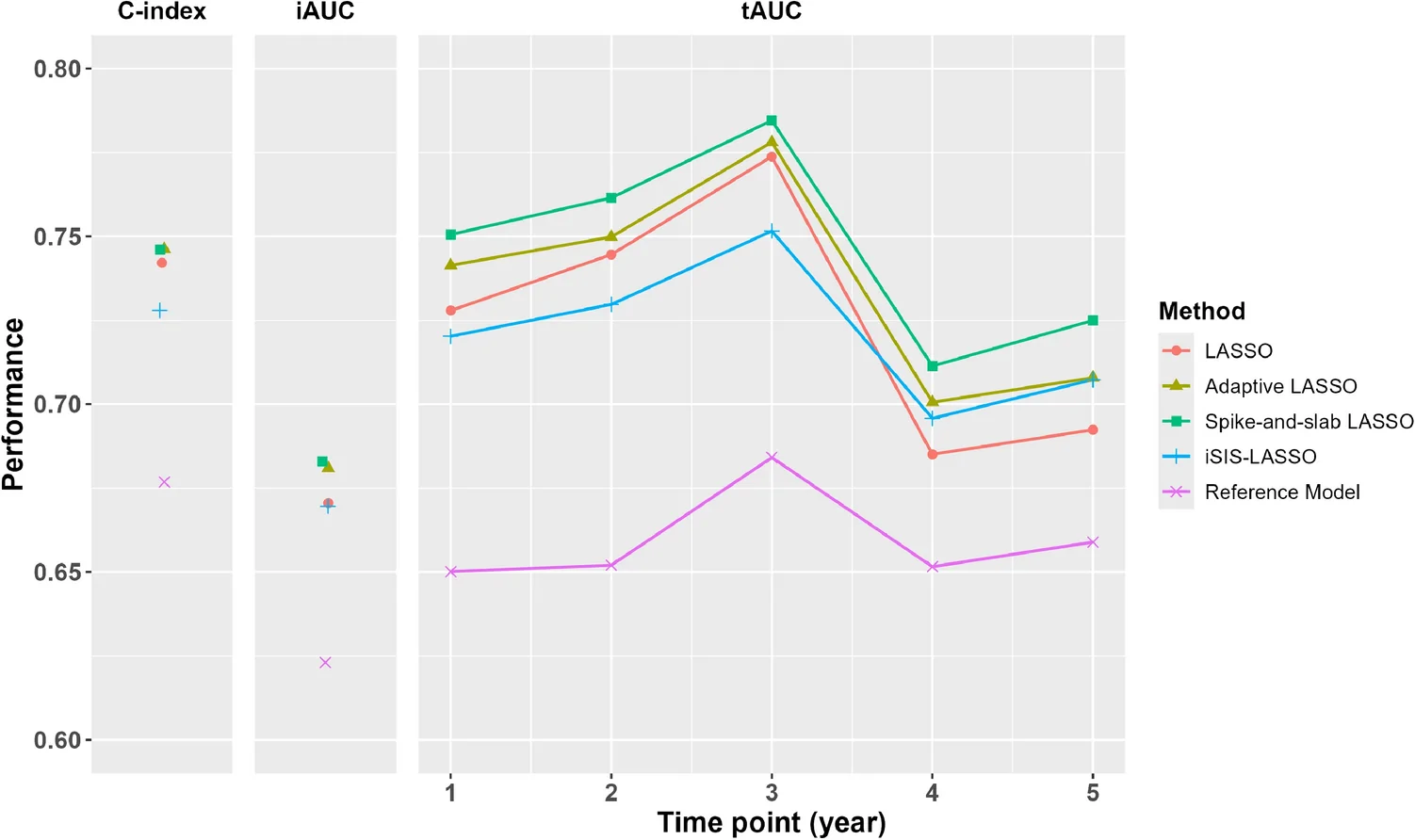
Discrimination performance of models. Performance metrics were evaluated using nested cross-validation. Mean performance across outer folds is shown for concordance index (C-index; left panel), integrated AUC (iAUC; middle panel) over the entire follow-up period, and the time-dependent AUC (tAUC; right panel) at $1^{st}, 2^{nd}, 3^{rd}, 4^{th}, \text {and}\ 5^{th}$ year. The models under comparison include LASSO, adaptive LASSO, spike-and-slab LASSO, iSIS-LASSO, and a clinical reference model. The corresponding performance is distinguished by different colors and marker shapes. Higher values indicate better discrimination performance.

All metabolomics-based penalized models demonstrated higher mean nested cross-validation estimates of discrimination over the reference model (Fig. 1). C-indices were 0.74 for LASSO, 0.75 for adaptive LASSO, 0.75 for spike-and-slab LASSO, 0.73 for iSIS-LASSO, compared with 0.68 for the reference model. A consistent pattern was observed for iAUC: spike-and-slab LASSO and adaptive LASSO had the highest iAUC (0.68). Time-dependent AUCs across the 5-year horizon showed how discrimination changed over follow-up time. The performance peaked at year 3 for all penalized models (LASSO: 0.77, adaptive LASSO: 0.78, spike-and-slab LASSO: 0.79, iSIS-LASSO: 0.76), compared with 0.68 for the reference model. Although tAUCs declined after 3 years of follow-up, the penalized models, particularly spike-and-slab and adaptive LASSO, maintained higher discrimination than the reference at every time point. The 95% CIs indicated considerable uncertainty in the estimated discrimination performance across all models (Supplement Table S1.3 and Table S1.5). For the iAUC, the lower bounds of the CIs for all metabolomics-based penalized models remained above 0.5, whereas the reference model’s CI extended below 0.5. Although discrimination estimates were consistently higher for the metabolomics-based penalized models, the estimated improvements over the reference model were modest, with increases of 0.05–0.07 for the C-index, 0.05–0.06 for the iAUC (Supplement Table S1.4), and 0.03−0.11 across follow-up time points for tAUC (Supplement Table S1.6). The bootstrap-based 95% CIs for the estimated improvements over the reference model included zero for all metabolomics-based models.

### Final models and model calibration

For all models, tumor stage (stage I–III *vs.* stage IV as the reference) and age at diagnosis were included as clinical covariates. Tumor stage showed consistently large effects, with lower hazard for patients with stage I–III *vs.* stage IV (${\hat{\beta }}_{\text {median}} \approx -2.09$; range *-2.12* to *-2.08*; median and range denote the median and minimum-to-maximum coefficient estimates across models), whereas age at diagnosis showed a small positive effect overall (${\hat{\beta }}_{\text {median}} \approx 0.005$; range *-0.003* to 0.010), suggesting slightly higher risk with increasing age (Fig. 3, Table 2). Several metabolites showed reproducible and directionally consistent associations with OS. In particular, 2-hydroxyisobutyrate was selected by all penalized models with negative coefficients (${\hat{\beta }}_{\text {median}} \approx -0.23$; range *-0.43* to *-0.02*), indicating a potentially inverse association with mortality. In contrast, indolelactate (${\hat{\beta }}_{\text {median}} \approx 0.31$; range 0.13 to 0.58) and a uridine-like metabolite (${\hat{\beta }}_{\text {median}} \approx 0.31$; range 0.24 to 1.00) were consistently selected with positive coefficients, suggesting that higher levels of these metabolites are associated with increased hazard.

**Fig. 2:**
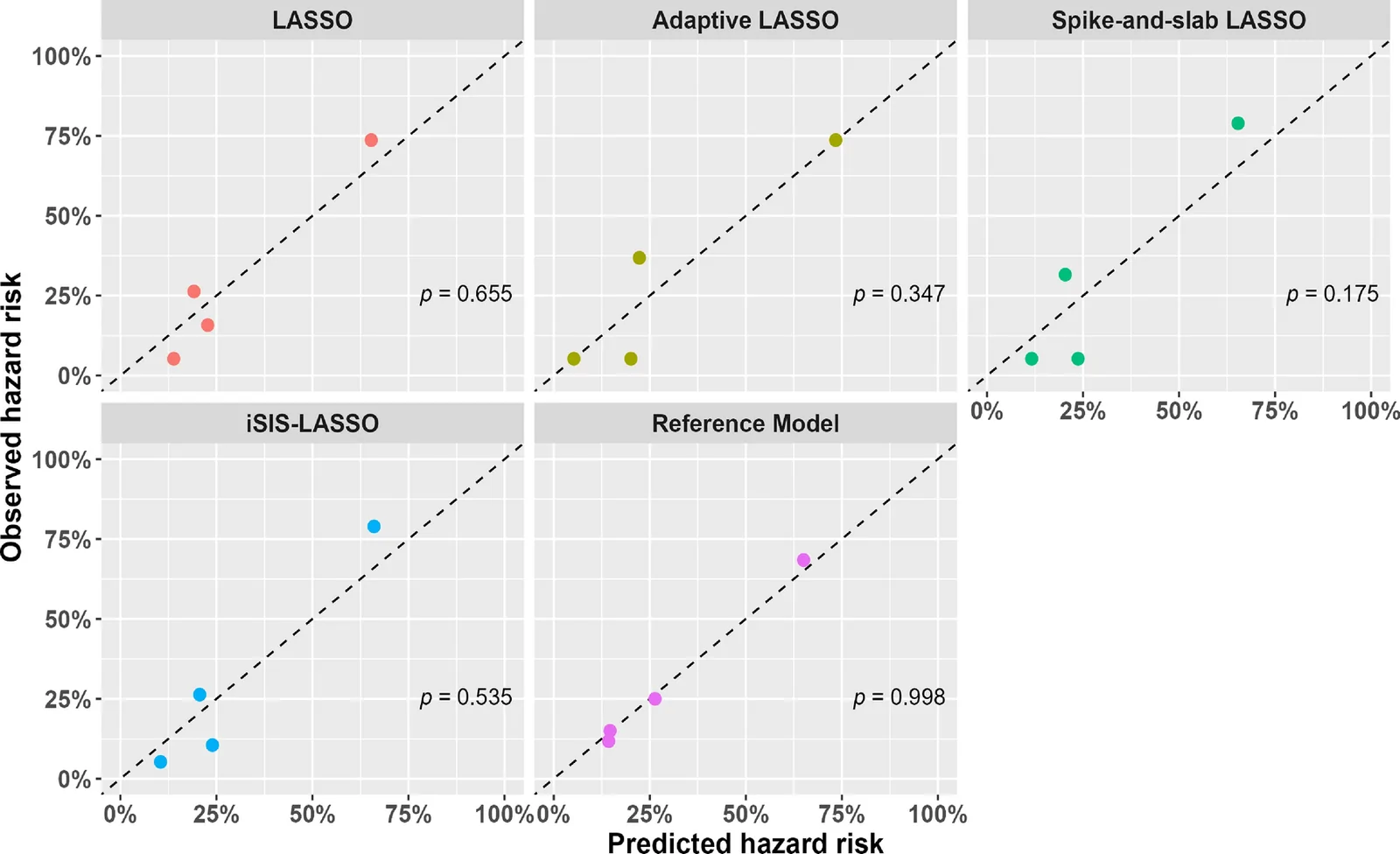
Calibration assement of final prognostic models fit on all study participants. Each panel corresponds to a modeling approach (LASSO, adaptive LASSO, spike-and-slab LASSO, iSIS-LASSO, or the clinical reference model) and displays the observed versus predicted hazard risk across patient risk groups. Risk groups were defined by quartiles of the model-specific risk score (linear predictor). The dashed diagonal line represents perfect calibration. The p-value shown in each panel is from a goodness-of-fit test comparing the predicted and observed risks across the quartiles of predicted risk. A p-value *< 0.05* indicates poor calibration.

For model calibration, we compared observed and predicted event rates across risk quartiles defined by the linear predictor (Fig. 2). Calibration was summarized using the observed-to-expected (O/E) ratio of observed to model-predicted event counts. Across models, O/E values were generally close to 1 across quartiles, indicating good agreement between observed and predicted event rates (Table 3). Most models showed mild overprediction in the lowest-risk quartile (O/E *< 1*), and near agreement or slight underprediction in the highest-risk quartile (e.g., O/E *≈ 1.12*). In the Poisson calibration models, likelihood ratio tests for group effects yielded *p*-values of 0.66 (LASSO), 0.35 (adaptive LASSO), 0.18 (spike-and-slab LASSO), 0.54 (iSIS-LASSO), and 1.00 (reference Cox model), indicating no evidence that observed event counts differed from model-expected counts across quartiles.

### Sensitivity analysis

In sensitivity analyses additionally adjusting for tumor site, model discrimination and calibration were comparable to those observed in the primary analyses (see Supplement 2). The composition of the final models remained stable, with key metabolites, particularly the uridine-like metabolite and indolelactate, consistently selected with similar effect estimates across models.

### Simulation study

Simulation analyses showed that, across all scenarios (varying signal strength, multicollinearity and censoring rates), the four modeling procedures demonstrated comparable discrimination performance. However, clear trade-offs emerged in variable selection and parameter estimation. Notably, iSIS-LASSO model achieved the highest rate of true signal recovery (full-set recovery), but also exhibited the largest $\ell _2$-Norm of model coefficient estimation error among the penalized models (see Supplement 3).

### Metabolite enrichment set analysis

The core metabolite lists (2-hydroxyisobutyrate(HMDB0000729), indolelactate(HMDB0000671), and uridine(HMDB0000296) as a proxy for the uridine-like metabolite) yielded enrichment of two disease-associated metabolite sets, “Argininemia” and “Colorectal cancer”, however, the enrichment sets did not meet the FDR threshold of 0.05 after adjusted for multiple comparisons (Fig. S1.1). Using the spike-and-slab LASSO model-derived metabolite list (2-hydroxyisobutyrate, valine (HMDB0000883), xylitol(HMDB0242149), indolelactate, uridine), we observed nominal enrichment across several disease-metabolite sets, most prominently for “Colorectal cancer”. After adjusting for multiple comparisons, these enrichments did not meet the FDR threshold of 0.05. (Fig. S1.2).

## Discussion

We evaluated whether preoperative urinary metabolites could improve CRC OS prediction beyond tumor stage and age at diagnosis. As expected, tumor stage remained the dominant prognostic factor, whereas age showed a small effect. In addition to these clinical covariates, several urinary metabolites contributed to prognostic prediction. Across four penalized Cox models (LASSO, adaptive LASSO, spike-and-slab LASSO, and iSIS-LASSO), variable selection showed highly concordance, with comparable coefficient estimates, and 2-hydroxyisobutyrate, indolelactate, and a uridine-like metabolite were consistently identified as predictors of CRC OS.

In nested cross-validation, metabolite-augmented penalized models achieved higher point estimate of C-indices, iAUCs and tAUCs (at all the evaluated time points), compared to the stage- and age-only reference model. However, bootstrap analyses yielded relatively wide 95% CIs for the discrimination estimates, while the CIs for the estimated improvements over the reference model included zero for all discrimination metrics. Both findings indicate the uncertainty of these estimates and likely reflect the limited sample size and number of observed events. Also, we cannot determine whether the observed incremental prognostic information is sufficient to alter clinical risk stratification, treatment selection, or patient management. Because the present study was designed to evaluate incremental prognostic information beyond age and tumor stage rather than clinical utility. Such assessments will require dedicated decision-focused analyses, including evaluation of clinically relevant risk thresholds, risk reclassification, decision-curve analysis, and net clinical benefit.

For the final model, calibration analyses showed reasonable agreement between observed and model-predicted events. O/E ratios close to 1 indicated that the observed number of deaths was generally consistent with the number expected under the model within each risk stratum, while non-significant Poisson calibration tests suggested no evidence of systematic over- or underprediction of risk. The final model coefficients showed consistent directions of association across penalized models, although their magnitudes varied. In particular, 2-hydroxyisobutyrate was consistently associated with lower hazard, with coefficients ranging from − 0.43 to − 0.02, whereas indolelactate and the uridine-like metabolite were consistently associated with higher hazard, with coefficients ranging from 0.13 to 0.58 and 0.24 to 1.00, respectively. These findings suggest that robustness in the present study is better reflected by the repeated selection of the same metabolites and the consistency of their directions of association than by the exact magnitude of individual coefficients.

The simulation study evaluated predictive discrimination across four penalized models while preserving the empirical correlation structure of the metabolomic data. Moderate discrimination (C-index > 0.70) was achievable under stronger signal scenarios (e.g., *β* = 0.7), even with high censoring, supporting the performance observed when metabolites were added to the clinically adjusted model. While the discrimination performance was similar across penalized models, differences emerged in variable selection and estimation. Specifically, iSIS-LASSO recovered more true signals but with larger model coefficient estimation error. This trade-off is consistent with known challenges of penalized regression in high-dimensional settings, where shrinkage can introduce bias in coefficient estimates (Tibshirani, 1996; Fan & Lv, 2008). Importantly, several metabolites selected by iSIS-LASSO were consistently identified by the other penalized methods, strengthens the evidence that overlapping features represent robust biological signals rather than method-specific artifacts. In practice, these findings suggest that the choice of penalized method depends on the analytic goal. Adaptive LASSO and spike-and-slab LASSO may be preferable when emphasis is placed on overall prediction performance and parsimonious model selection, whereas iSIS-LASSO may be advantageous when prioritizing the retention of potentially important predictors, albeit at the cost of less stable coefficient estimation and greater susceptibility to false positives.

Several metabolite signals were consistently associated with OS across models, but their annotation confidence differed. The identified panel included confidently annotated metabolites (Level 1) such as 2-hydroxyisobutyrate, indolelactate, valine, and xylitol, as well as partially annotated features including a uridine-like and sugar-acid features lacking unique compound-level annotation (Level 3). Accordingly, these latter signals warrants cautious biological interpretation, as the associations with OS were consistent across models but the Level 3 annotation limits definitive conclusions regarding the underlying compounds or pathways. These metabolites are involved in biological processes related to branched-chain amino acid and energy metabolism (2-hydroxyisobutyrate and valine) (Xu et al., 2023; Zhou et al., 2025b; Brown et al., 2026; Huang et al., 2018), microbial tryptophan–-indole metabolism (indolelactate) (Liu et al., 2025), carbohydrate and polyol metabolism (xylitol and a sugar-acid feature) (Cannon et al., 2025), and nucleoside turnover (uridine) (Choi et al., 2025). These biological interpretations are based on the known functions and previously reported associations of the individual metabolites. The identified metabolite panels yielded nominal enrichment for several disease-associated metabolite sets, including colorectal cancer; however, none of these enrichments remained statistically significant after FDR correction. These pathway-level enrichment findings should therefore be interpreted cautiously and viewed as exploratory.

In our study, higher urinary 2-hydroxyisobutyrate (2-HIB) and valine were associated with better OS. Previous CRC studies have reported mixed findings regarding valine and branched-chain amino acid (BCAA) metabolism (Brown et al., 2026; Xu et al., 2023; Zhou et al., 2025b), whereas evidence on urinary 2-HIB remains scarce. To our knowledge, no studies have evaluated urinary 2-HIB in relation to cancer survival. Although 2-HIB and 3-hydroxyisobutyrate (3-HIB) share the same molecular formula, they are distinct structural isomers with different biological functions. Unlike 2-HIB, 3-HIB is a well-characterized intermediate of mitochondrial valine catabolism that is generated by the hydrolysis of 3-hydroxyisobutyryl-CoA catalyzed by 3-hydroxyisobutyryl-CoA hydrolase (HIBCH). Shan et al. (2019) demonstrated that increased HIBCH expression promotes CRC growth using analyses of human CRC tissues, in vitro cell-line experiments, and mouse xenograft models. Because 2-HIB is not part of this valine catabolic pathway, its association with better OS may reflect biological processes distinct from those described for 3-HIB.

Differences in biospecimen type (urine versus tumor tissue) and biological context may also partly explain the discrepancies between previous studies and our findings. As highlighted by Brown et al. (2026), the role of BCAA metabolism in cancer is highly context dependent, with tumor-intrinsic metabolic requirements differing from systemic host metabolism. While tumor cells utilize valine to support proliferation, lower systemic amino acid availability may reflect malnutrition, cancer cachexia, or a more depleted metabolic state (Xu et al., 2023; Zhou et al., 2025b). Thus, higher urinary valine may indicate a more preserved host metabolic status rather than greater tumor-promoting activity, potentially contributing to better survival.

Higher urinary indolelactate, also known as indole-3-lactic acid (ILA), was associated with increased hazard in our study. This finding contrasts with previous studies describing ILA as a locally anti-inflammatory and potentially tumor-suppressive gut microbial tryptophan metabolite (Liu et al., 2023; Zhou et al., 2025a). Prior experimental work demonstrated that exogenous ILA suppressed CRC development and tumor growth in vitro and in azoxymethane/dextran sodium sulfate (AOM/DSS)-induced and xenograft mouse models (Zhou et al., 2025a). However, these findings are not directly comparable with our survival analysis because they focused on intestinal tryptophan metabolism, including fecal metabolomics, comparisons between patients with CRC and healthy controls, and experimental models of tumor development. In contrast, our study examined urinary metabolites in patients with established CRC in relation to OS. Therefore, higher urinary ILA may reflect systemic metabolism or metabolite excretion rather than local intestinal tryptophan metabolism (Liu et al., 2025).

Higher urinary xylitol was also associated with increased hazard in our study. This appears to contrast with experimental work suggesting potential anti-tumor effects of xylitol under selected conditions (Cannon et al., 2025; Sahasakul et al., 2022). In syngeneic mouse cancer models, Cannon et al. (2025) reported reduced tumor growth or improved survival under selected experimental conditions. Similarly, replacement of glucose with xylitol prolonged survival in an orthotopic xenograft model of tongue cancer (Sahasakul et al., 2022). These experimental findings reflect direct xylitol exposure in controlled model systems, whereas our study measured urinary xylitol concentrations in humans. Higher urinary xylitol should therefore not be interpreted as evidence of a direct harmful effect. Rather, it may represent an excretion-based metabolomic signal related to dietary exposure, altered carbohydrate or polyol metabolism, metabolic risk, or other host factors associated with cancer survival.

The uridine-like metabolite showed an association with poorer survival, which is consistent with reports linking altered uridine metabolism to increased RNA turnover and tumor cell proliferation in cancer (Choi et al., 2025; Rais et al., 2024). However, because this metabolite was assigned annotation Level 3 (putatively characterized compound class), its biological interpretation should be considered tentative.

Several limitations should be acknowledged. The limited sample size (23 deaths among 76 patients) relative to the number of candidate predictors increases the risk of overfitting and coefficient instability, even though penalized estimation and simulation analyses were used to enhance the internal robustness, and nested cross-validation was applied to reduce over-optimism. These methods, however, do not replace independent external validation. Accordingly, the identified metabolite signatures should be interpreted as exploratory and hypothesis-generating rather than definitive prognostic markers. Also, the small sample size limits model complexity. Some potentially important prognostic factors, such as treatment history, lifestyle, comorbidities and Eastern Cooperative Oncology Group (ECOG) performance status, were not included in the current models to minimize model complexity and the risk of overfitting. Therefore, it limits the reference model’s scope to only considering the stage and age at diagnosis. Future studies with larger sample sizes and more events should evaluate the incremental performance of incorporating extended clinical factors. Furthermore, the analytical depth was constrained by the technology available at the time of sample acquisition. The single-quadrupole GC-MS instrument necessitated a trade-off between scan rates and dwell times, limiting sensitivity and metabolome coverage compared to modern GC-TOF-MS instruments. In addition, MSEA was performed on a parsimonious set of 3–5 metabolites. This small input size limits statistical power, increasing the risk of false-positive overrepresentation.

## Conclusions

This study demonstrates that preoperative urinary metabolites may improve CRC OS prediction beyond tumor stage and age at diagnosis. Across metabolomics-based penalized models, a small metabolite panel, including indolelactate, 2-hydroxyisobutyrate, and a uridine-like metabolite, emerged as robust signals linked to relevant metabolic and microbiome-related pathways.

A complementary simulation study, leveraging the empirical metabolite correlation structure, suggested that these findings are consistent with metabolite effects of limited magnitude under realistic sample size and censoring conditions. However, this study was limited by the small sample size and low number of events. Accordingly, these findings should be considered exploratory and hypothesis-generating. Studies examining urinary metabolomics and OS in CRC remain scarce, our results provide initial evidence that urinary metabolites may have prognostic potential and warrant validation in larger, independent multicenter cohorts.

**Fig. 3:**
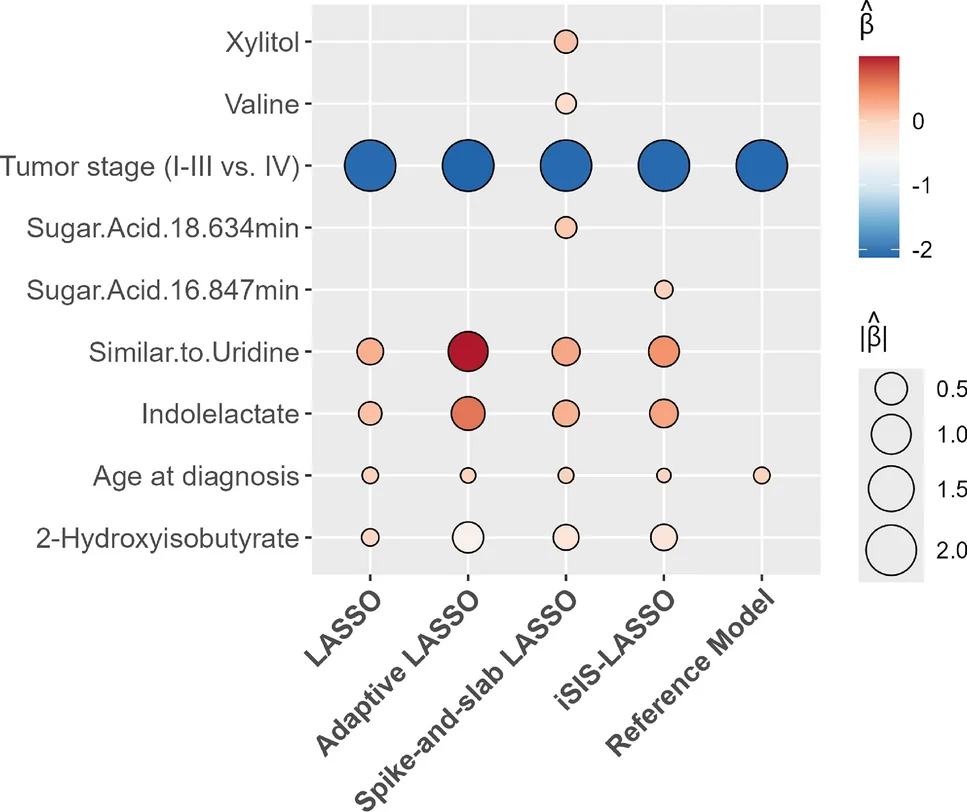
Model coefficients of final prognostic models fit on all study participants. Each column corresponds to a modeling approach (LASSO, adaptive LASSO, spike-and-slab LASSO, iSIS-LASSO or the clinical reference model), and each row represents a clinical or metabolite feature. Features identified in the corresponding final model are displayed as bubbles. Bubble color represents the sign and magnitude of the estimated log-hazard ratio (${\hat{\beta }}$), with red indicating positive associations and blue indicating negative associations. Bubble size is proportional to the absolute value of the estimated log-hazard ratio ($|{\hat{\beta }}|$).

**Table 1:** Patient characteristics at baseline

	Overall (N = 76)
Age at diagnosis	
Mean (SD)	65.4 (12.3)
Median [Min, Max]	66.0 [35.0, 90.0]
Sex	
Female	26 (34.2%)
Male	50 (65.8%)
Tumor site	
Colon	35 (46.1%)
Rectum	41 (53.9%)
Tumor stage	
I	11 (14.5%)
II	26 (34.2%)
III	23 (30.3%)
IV	16 (21.1%)
Neoadjuvant Treatment	
No	51 (67.1%)
Yes	25 (32.9%)
Adjuvant Treatment	
No	48 (63.2%)
Yes	28 (36.8%)

**Table 2:** Model coefficients for selected features in final models*

Feature	LASSO	Adaptive LASSO	Spike-and-slab	iSIS-LASSO	Reference model
Xylitol	–	–	0.124	–	–
Valine	–	–	− 0.061	–	–
Tumor stage (I-III vs. IV)	− 2.084	− 2.123	− 2.085	− 2.088	− 2.098
Sugar.Acid.18.634min	–	–	0.083	–	–
Sugar.Acid.16.847min	–	–	–	0.025	–
Similar.to.Uridine	0.235	0.999	0.291	0.419	–
Indolelactate	0.130	0.583	0.229	0.313	–
Age at diagnosis	0.009	−0.003	0.005	0.002	0.010
2-Hydroxyisobutyrate	− 0.017	− 0.433	− 0.208	− 0.232	–

*The reference model includes only tumor stage and age at diagnosis; penalized models are adjusted for tumor stage and age at diagnosis.

**Table 3:** Calibration assessment of the final models using observed and expected event rates across predicted risk quartiles

Method	Quartile 1 (O/E*)	Quartile 2 (O/E*)	Quartile 3 (O/E*)	Quartile 4 (O/E*)
LASSO	0.053/0.139	0.158/0.227	0.263/0.191	0.737/0.653
Adaptive LASSO	0.053/0.052	0.053/0.201	0.368/0.223	0.737/0.734
Spike-and-slab LASSO	0.053/0.116	0.053/0.237	0.316/0.204	0.789/0.654
iSIS-LASSO	0.053/0.105	0.105/0.239	0.263/0.206	0.789/0.660
Reference Model	0.150/0.147	0.250/0.264	0.118/0.143	0.684/0.650

*O/E denotes the ratio of observed (O) and expected (E) event rates within each predicted risk quartile; E was computed as the mean predicted event probability in that quartile.

## Supplementary Information

Below is the link to the electronic supplementary material.

Supplementary file 1 (pdf 3550 KB)

## Data Availability

The ColoCare Study data are available from colocarestudy_admin@hci.utah.edu on reasonable request and as described on the ColoCare website (https://uofuhealth.utah.edu/huntsman/labs/colocare-consortium/). Our data sharing procedures are available online (https://uofuhealth.utah.edu/huntsman/labs/colocare-consortium/data-sharing/new-projects.php). For any additional questions, please contact the ColoCare Study Administrator Team (colocarestudy_admin@hci.utah.edu).
