# Supplementary material for: Urinary metabolomics may improve prediction of overall survival beyond tumor stage in colorectal cancer: results from the ColoCare study
Source: Metabolomics. 2026 Sep 11;22(5):150. doi: 10.1007/s11306-026-02516-3 (PMC13569605; doi:10.1007/s11306-026-02516-3)
Supplement: Supplementary file 1 [file 11306_2026_2516_MOESM1_ESM.pdf]

Supplementary Material

Supplement 1a: Model Specification & Model Calibration

Table S1.1 summarizes the penalized Cox regression approaches used in this study, including LASSO, adaptive LASSO, spike-and-slab LASSO, and iSIS-LASSO. For each model, the table outlines the penalty structure, variable selection strategy, tuning procedure, and treatment of clinical covariates, highlighting the key distinctions in how high-dimensional metabolite data were incorporated into the models.

Table S1.2 provides a stepwise overview of the Poisson regression model-based calibration procedure. For each subject, the cumulative hazard over follow-up was computed, which under the Cox model corresponds to the expected number of events. These quantities were then aggregated within risk strata defined by the linear predictor. A Poisson regression framework was used to formally evaluate goodness-of-fit while appropriately accounting for censoring.

Table S1.1. Model specifications of penalized Cox modeling

Model	Description
**LASSO**	An ℓ_1-penalized Cox proportional hazards model was fit including all metabolites together with tumor stage and age at diagnosis. Tumor stage and age were treated as unpenalized covariates, and penalization was applied only to metabolite coefficients. The penalty parameter λ was selected using stratified K-fold cross-validation, choosing the value that minimized the cross-validated partial likelihood deviance ("lambda.min").
**Adaptive LASSO**	A two-step procedure was used. First, a standard LASSO Cox model was fit to obtain initial metabolite coefficient estimates $\tilde{\beta}_j$. Second, a weighted LASSO Cox model was fit using penalty weights $w_j = \frac{1}{\lvert\tilde{\beta}_j\rvert^\gamma}, \quad \gamma = 1$, applied to metabolite coefficients. Tumor stage and age at diagnosis were unpenalized in both steps. The penalty parameter was selected using the same cross-validation procedure as in the standard LASSO.
**Spike-and-slab LASSO**	A spike-and-slab LASSO Cox model was fit, assigning metabolite coefficients a two-component spike–slab prior with fixed scales $ss = c(0.15, 0.5)$. Estimation proceeded via an EM algorithm, updating prior scales and inclusion probabilities and re-estimating coefficients using variable-specific penalties. Tumor stage and age at diagnosis were kept unpenalized by assigning them large prior scales and excluding them from spike–slab updates. The model was implemented using internal routines from the BhGLM R package (Yi et al., 2019), with minimal modifications to `.bmlasso()` and `.bmlasso.fit()` to allow adjusting clinical covariates.
**iSIS-LASSO**	An iterative SIS–LASSO Cox procedure with the conservative (intersection) variant was applied (Fan et al., 2009), consisting of repeated cycles of covariate-adjusted screening and penalized model fitting. At each iteration, the sample was randomly split into two halves, and metabolites were ranked within each half using partial likelihood from Cox models adjusted for tumor stage and age at diagnosis; previously selected metabolites were included as conditioning variables from the second iteration onward. The top m metabolites from each half were selected, increasing m until their intersection contained $d = \left\lfloor \frac{n}{\log(n)} \right\rfloor$, with smaller values (e.g. $d = \left\lfloor \frac{n}{2\log(n)} \right\rfloor$,) used if convergence issues arose (De Bin et al., 2020; Jardillier et al., 2022). The intersected metabolites were then entered into a LASSO Cox model (tumor stage and age at diagnosis unpenalized), and iterations (up to 50) continued until the selected set stabilized or the target size was reached.

Table S1.2. Calibration assessment procedure

**Step**	**Description**
**(i) Individual-level expected event estimation**	For each final model, we computed individual risk scores, using the linear predictor (lp_i). To estimate expected event incidences, we utilized the hazard function $h_i(t)$, which represents the instantaneous rate of event occurrence at time t conditional on survival up to that time. Specifically, we fit an offset Cox model, $h_i(t) = h_0(t)\exp(lp_i)$, where the relative risk $\exp(lp_i)$ remained fixed to allow for the estimation of the baseline hazard $h_0(t)$. This was implemented using the R command `survival::coxph(y ~ offset(lp))`. Based on this fit, we calculated the expected event incidences e_i for each subject, equivalent to the cumulative hazard over their respective follow-up period, using `stats::predict(fit, type = "expected")`.
**(ii) Risk grouping**	Subjects were ranked according to lp_i and divided into quartiles. Quartiles were chosen to reflect the limited number of observed events while ensuring sufficient expected and observed counts within each group.
**(iii) Group-level observed and expected counts**	Within each risk group g, observed event counts were calculated as $$O_g = \sum_{i \in g} \delta_i,$$ where δ_i is the event indicator. Group-level expected counts were obtained by aggregating individual expected values: $$E_g = \sum_{i \in g} e_i.$$ Under perfect calibration, $O_g \approx E_g$ (i.e., $O_g/E_g \approx 1$).
**(iv) Poisson calibration modeling and goodness-of-fit test**	Calibration was formally evaluated using a Poisson regression model $$O_g \sim \text{Poisson}(\mu_g), \quad \log(\mu_g) = \log(E_g) + \gamma_g,$$ with $\log(E_g)$ included as an offset (Atkinson et al., 2008; Berry, 1983). In R, this was implemented using `glm(status ~ -1 + group + offset(log(e)), family="poisson")`. <br>In this parameterization, $\exp(\gamma_g) = O_g/E_g$ represents the group-specific calibration factor (or standardized incidence ratio). Values equal to 1 indicate perfect calibration; values greater than or less than 1 indicate systematic underestimation or overestimation of risk, respectively. <br>Overall calibration was assessed using a likelihood ratio goodness-of-fit test comparing models with and without group effects. A non-significant p-value indicates no evidence of systematic miscalibration across risk strata (Crowson et al., 2016).

Supplement 1b: Uncertainty quantification of discrimination performance across modeling strategies

Table S1.3. Nested cross-validation estimate of global discrimination performance with bootstrap 95% confidence intervals (1,000 bootstrap repetitions).

Method	C-index*	iAUC**
LASSO	0.74 (0.64, 0.92)	0.67 (0.59, 0.95)
Adaptive LASSO	0.75 (0.63, 0.92)	0.68 (0.58, 0.94)
Spike-and-slab LASSO	0.75 (0.66, 0.90)	0.68 (0.59, 0.94)
iSIS-LASSO	0.73 (0.66, 0.93)	0.67 (0.59, 0.96)
Reference	0.68 (0.53, 0.84)	0.62 (0.49, 0.88)

* C-index = concordance index.
** iAUC = integrated area under the ROC curve.

Table S1.4. Nested cross-validation estimate of global discrimination improvement over the reference model with bootstrap 95% confidence intervals (1,000 bootstrap repetitions).

Method	ΔC-index*	ΔiAUC**
LASSO	0.07 (-0.05, 0.30)	0.05 (-0.07, 0.29)
Adaptive LASSO	0.07 (-0.08, 0.30)	0.06 (-0.08, 0.30)
Spike-and-slab LASSO	0.07 (-0.04, 0.26)	0.06 (-0.06, 0.27)
iSIS-LASSO	0.05 (-0.06, 0.31)	0.05 (-0.09, 0.31)

* ΔC-index = difference in concordance index.
** ΔiAUC = difference in integrated area under the ROC curve.

Table S1.5. Nested cross-validation estimate of time-dependent AUC (tAUC) with bootstrap 95% confidence intervals (1,000 bootstrap repetitions).

	tAUC*				
Method	Year 1	Year 2	Year 3	Year 4	Year 5
LASSO	0.73 (0.50, 0.96)	0.74 (0.61, 0.96)	0.77 (0.66, 0.96)	0.69 (0.53, 0.99)	0.69 (0.49, 1.00)
Adaptive LASSO	0.74 (0.49, 0.96)	0.75 (0.60, 0.96)	0.78 (0.65, 0.96)	0.70 (0.52, 0.99)	0.71 (0.48, 0.99)
Spike-and-slab LASSO	0.75 (0.51, 0.93)	0.76 (0.61, 0.95)	0.78 (0.67, 0.96)	0.71 (0.51, 1.00)	0.72 (0.48, 1.00)
iSIS-LASSO	0.72 (0.52, 0.96)	0.73 (0.62, 0.96)	0.75 (0.67, 0.96)	0.70 (0.51, 1.00)	0.71 (0.49, 1.00)
Reference	0.65 (0.39, 0.90)	0.65 (0.48, 0.90)	0.68 (0.53, 0.90)	0.65 (0.46, 0.96)	0.66 (0.42, 0.96)

* tAUC = time-dependent area under the ROC curve.

Table S1.6. Nested cross-validation estimate of time-dependent AUC (tAUC) improvement over the reference model with bootstrap 95% confidence intervals (1,000 bootstrap repetitions).

	ΔtAUC*				
Method	Year 1	Year 2	Year 3	Year 4	Year 5
LASSO	0.08 (-0.09, 0.40)	0.09 (-0.08, 0.35)	0.09 (-0.06, 0.31)	0.03 (-0.12, 0.29)	0.03 (-0.12, 0.30)
Adaptive LASSO	0.09 (-0.12, 0.40)	0.10 (-0.10, 0.35)	0.09 (-0.09, 0.31)	0.05 (-0.14, 0.31)	0.05 (-0.14, 0.33)
Spike-and-slab LASSO	0.10 (-0.08, 0.38)	0.11 (-0.06, 0.32)	0.10 (-0.05, 0.30)	0.06 (-0.12, 0.28)	0.07 (-0.12, 0.28)
iSIS-LASSO	0.07 (-0.11, 0.40)	0.08 (-0.11, 0.36)	0.07 (-0.11, 0.32)	0.04 (-0.14, 0.34)	0.05 (-0.14, 0.34)

* ΔtAUC = difference in time-dependent area under the ROC curve.

Supplement 1c: Metabolite Set Enrichment Analysis

For metabolite set enrichment analysis, we constructed two model-specific input lists: 1) a core three–metabolite panel (2-hydroxyisobutyrate(HMDB0000729), indolelactate(HMDB0000671), uridine(HMDB0000296)), which includes metabolites that were repeatedly selected by the LASSO, adaptive LASSO, and iSIS–LASSO models; 2) the spike-and-slab LASSO model derived list (2-hydroxyisobutyrate, valine (HMDB0000883), xylitol(HMDB0242149), indolelactate, uridine). For enrichment analysis, uridine was used as a proxy for the uridine-like metabolite. Although this feature was annotated to Level 3 (putatively characterized compound class), its structural signature allowed for the selection of a representative identifier to facilitate mapping to established metabolic pathways. In contrast, two sugar acids (Sugar.Acid.16.847min and Sugar.Acid.18.634min) selected by the iSIS-LASSO and spike-and-slab LASSO models, respectively, were excluded from the MSEA. Unlike the uridine-like feature, these sugar acids lacked sufficient structural specificity to be assigned a unique representative identifier, making them ineligible for exact mapping in the MetaboAnalyst. The corresponding restults based on the two specific input lists are presented in **Figure S1.1** and **Figure S1.2**.

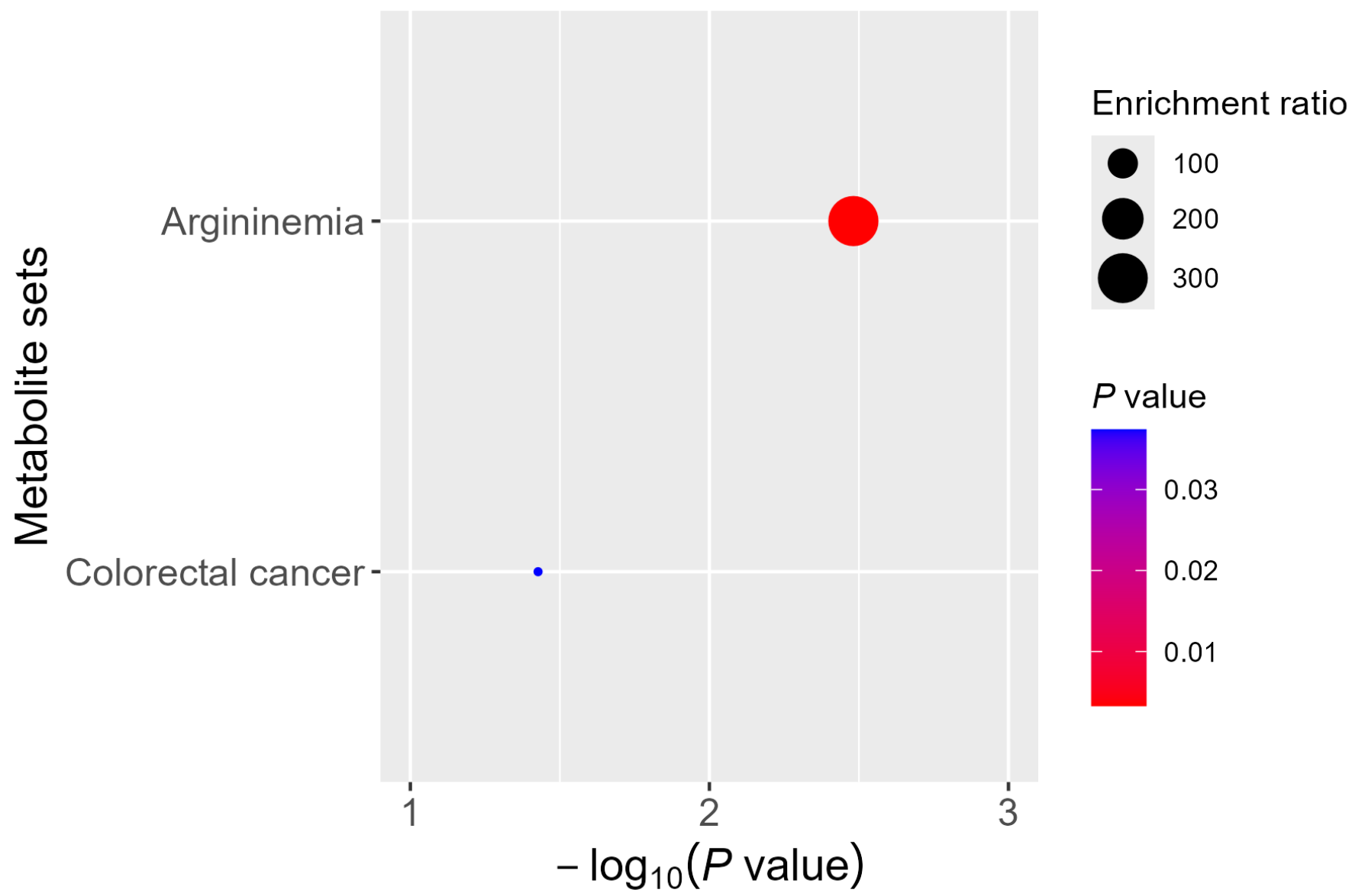

Figure S1.1. Metabolite set enrichment based on the core metabolite panel. Enrichment analysis was performed in MetaboAnalyst using the three metabolites (2-hydroxyisobutyrate, indolelactate, uridine) consistently selected by LASSO, adaptive LASSO, and iSIS–LASSO. An additional metabolite identified by iSIS–LASSO could not be mapped in MetaboAnalyst and was therefore excluded from the enrichment analysis. The x-axis shows $-log_{10}(P)$ and the y-axis lists enriched metabolite sets. Bubble size is proportional to the enrichment ratio and color indicates the nominal P value. This panel yielded nominal enrichment of “Argininemia” and “Colorectal cancer”, but neither remained significant after multiple-testing correction.

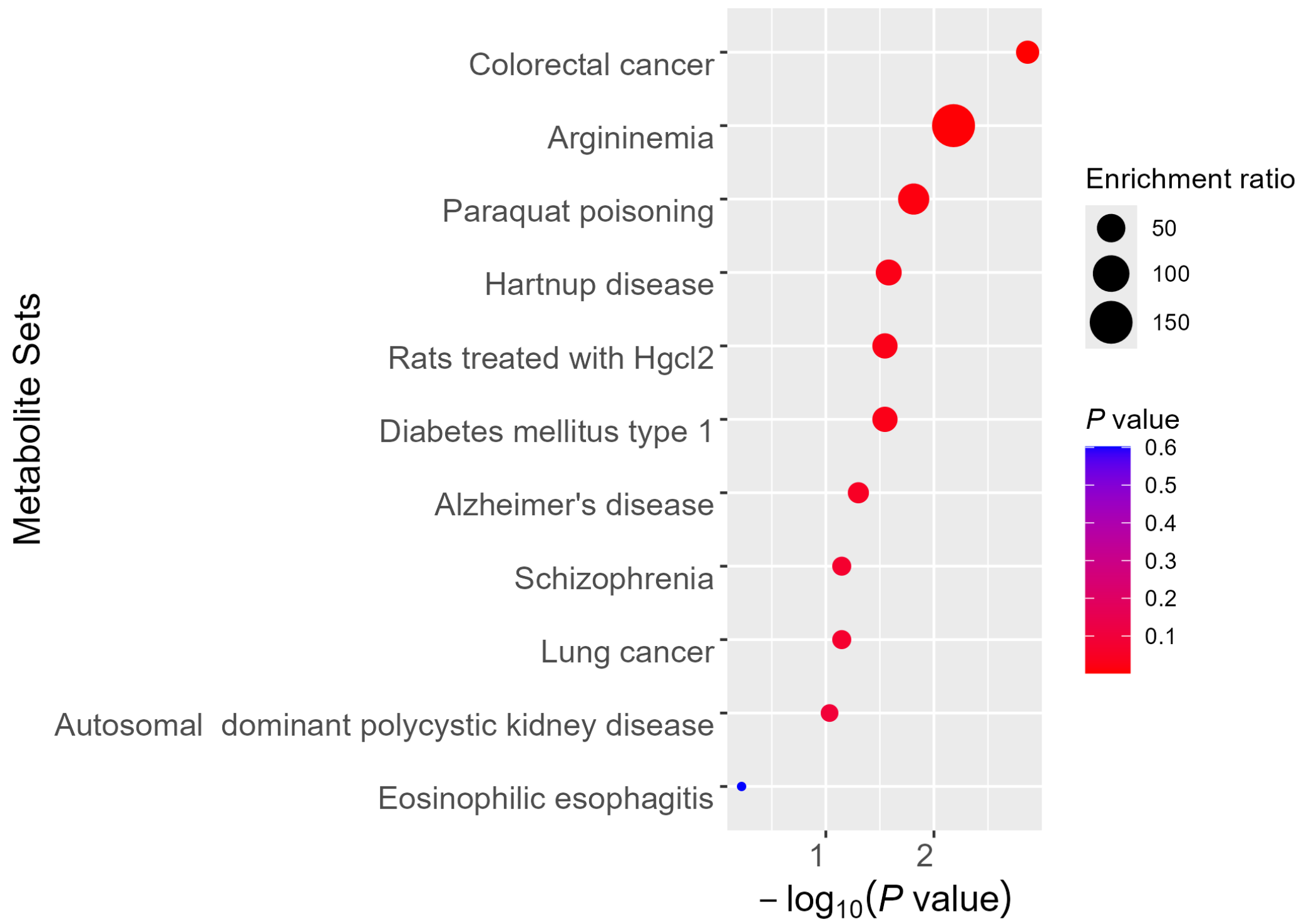

Figure S1.2. Metabolite-set enrichment based on the spike-and-slab LASSO derived metabolite panel. Enrichment analysis was conducted using the five metabolites identified by the spike-and-slab LASSO model (2-hydroxyisobutyrate, valine, xylitol, indolelactate, and uridine). The x-axis shows $-log_{10}(P)$ and the y-axis lists enriched metabolite sets. Bubble size reflects the enrichment ratio and color encodes the nominal P value. This panel showed broader but largely nominal enrichment across several disease-related sets including "Colorectal cancer", however, no sets remained significant after multiple-testing correction.

Supplement 2: Sensitivity Analyses

In sensitivity analysis, we evaluated model performance with additionally adjustment for tumor site (colon *vs.* rectum), given accumulating evidence suggesting an association between tumor site and OS in CRC. For this purpose, tumor site was included as an additional unpenalized covariate in the clinical reference model and in each of the four penalized Cox models. All other aspects of model specification, tuning, and validation procedures were identical to those used in the primary analyses.

Model discrimination

In both the primary and tumor site adjusted sensitivity analyses, all penalized models demonstrated better discrimination than the clinical reference model in terms of the C-index, iAUC and tAUC (Figure S2.1).

When ranking models based on overall discrimination metrics (C-index and iAUC), spike-and-slab LASSO and adaptive LASSO consistently performed among the top methods in both analyses, with only modest differences between penalized models. The reference model showed the lowest performance across both metrics.

When comparing tAUCs among the penalized models, we focused on summary characteristics rather than pointwise rankings at individual time horizons. Across both the primary and tumor site adjusted sensitivity analyses, all penalized models achieved comparable peak tAUC values, with spike-and-slab LASSO and adaptive LASSO generally attaining the highest peak discrimination, followed closely by standard LASSO and iSIS-LASSO. Differences in peak tAUC across penalized methods were modest. Similarly, average tAUC performance across follow-up periods was broadly comparable among the penalized models. Although the relative ordering of tAUCs varied at

specific follow-up times and between analyses, the overall magnitude and temporal patterns of tAUCs were similar across penalized models and consistently exceeded those of the reference model.

Final model calibration

Across both the primary and tumor site-adjusted sensitivity analyses, all models showed broadly similar calibration patterns, with observed and expected event rates increasing across predicted risk groups, and agreement observed across all risk groups (Figure S2.2, Table S2.2). Calibration was largely preserved after additional adjustment for tumor site, with no systematic deterioration across modeling approaches.

In sensitivity analysis additionally adjusting for tumor site, iSIS-LASSO model, calibration was assessed using a median split rather than quartiles because the risk scores derived from this model yielded insufficient distinct values to support stable quartile-based grouping. Observed and expected event rates in the resulting two risk groups were comparable, indicating no substantial loss of calibration.

Selected features and corresponding coefficients in the final models

Across both the primary and tumor site adjusted sensitivity analyses, tumor stage and age at diagnosis were included in all models and exhibited highly stable coefficient estimates across modeling approaches. Tumor stage consistently showed a strong association with OS, with nearly identical effect sizes before and after tumor site adjustment, while age at diagnosis demonstrated smaller, directionally consistent effects with minimal change in magnitude.

Among the penalized models, the core set of selected metabolites remained largely consistent

after adjustment for tumor site. A uridine-like metabolite and indolelactate were retained across all penalized models in both analyses, with only modest attenuation of their coefficient magnitudes after tumor site adjustment, suggesting limited confounding by tumor site. Metabolites such as xylitol and valine, which were selected by the spike-and-slab LASSO, also showed directionally consistent effects with slight attenuation after tumor site adjustment. Adjustment for tumor site led to the selection of a small number of additional metabolites in certain penalized models (e.g., Threonate, Tartrate, and Myo-inositol were selected by the iSIS-LASSO model), but these changes were model specific and did not substantially alter the dominant metabolomic signals or their directions of association.

Together, the stability of feature selection, coefficient estimates, and relative effect sizes before and after tumor-site adjustment supports the robustness of the identified metabolomic predictors and indicates that their associations with OS are largely independent of tumor site. Notably, the estimated effect of tumor site itself was modest across all models and substantially smaller than the effects of the consistently selected metabolites: a uridine-like metabolite and Indolelactate.

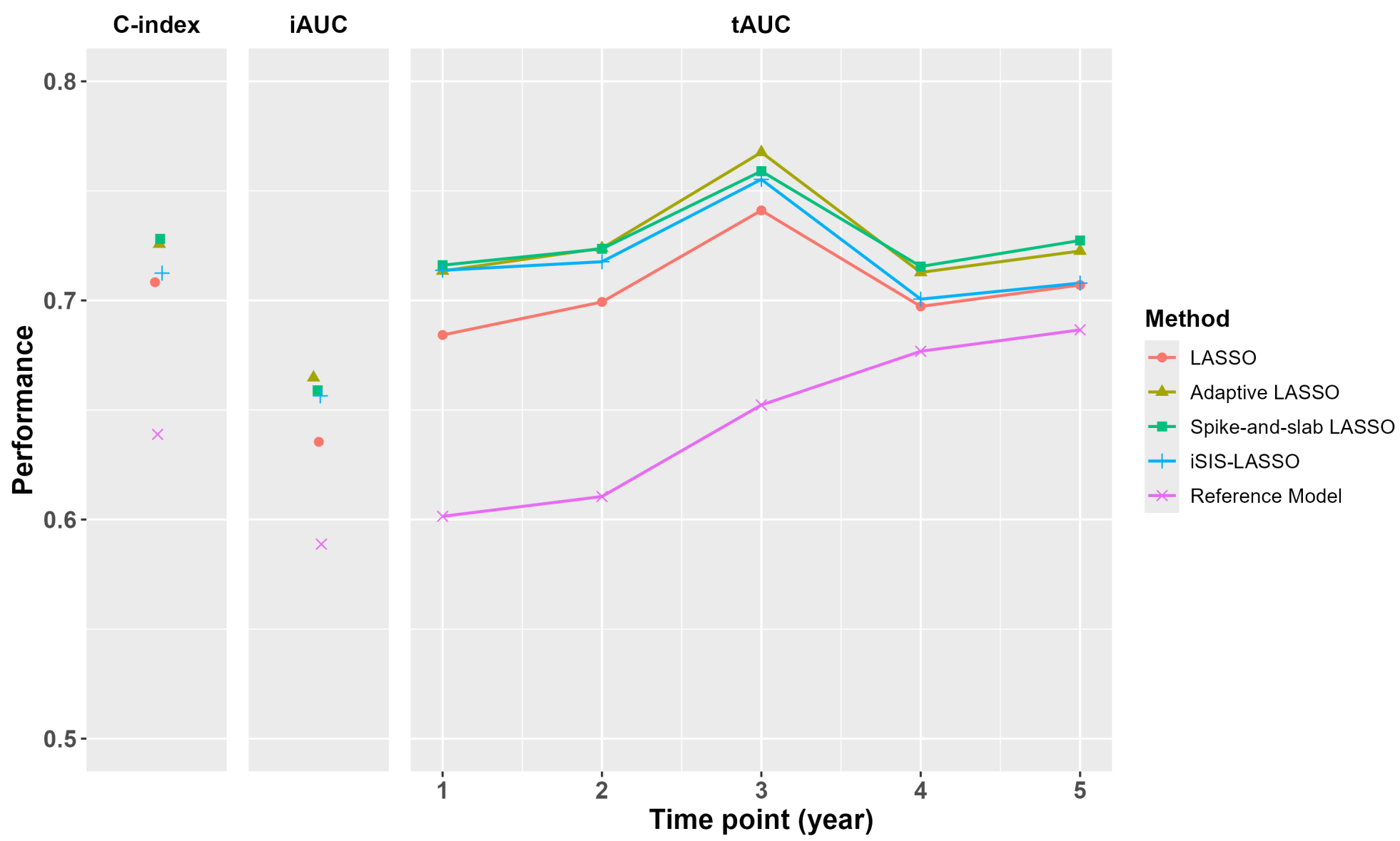

Figure S2.1. Model discrimination assessment (with additional adjustment for tumor site). Performance metrics were evaluated using nested cross-validation. Mean performance across outer folds is shown for concordance index (C-index; left panel), integrated AUC (iAUC; middle panel) over the entire follow-up period, and the time-dependent AUC (tAUC; right panel) at $1^{st}, 2^{nd}, 3^{rd}, 4^{th}$, and 5th year. The models under comparison include LASSO, adaptive LASSO, spike-and-slab LASSO, iSIS-LASSO, and a clinical reference model. The corresponding performance is distinguished by different colors and marker shapes. Higher values indicate better discrimination performance. All penalized models continue to outperform the clinical reference model and no substantive changes in relative model performance ranking.

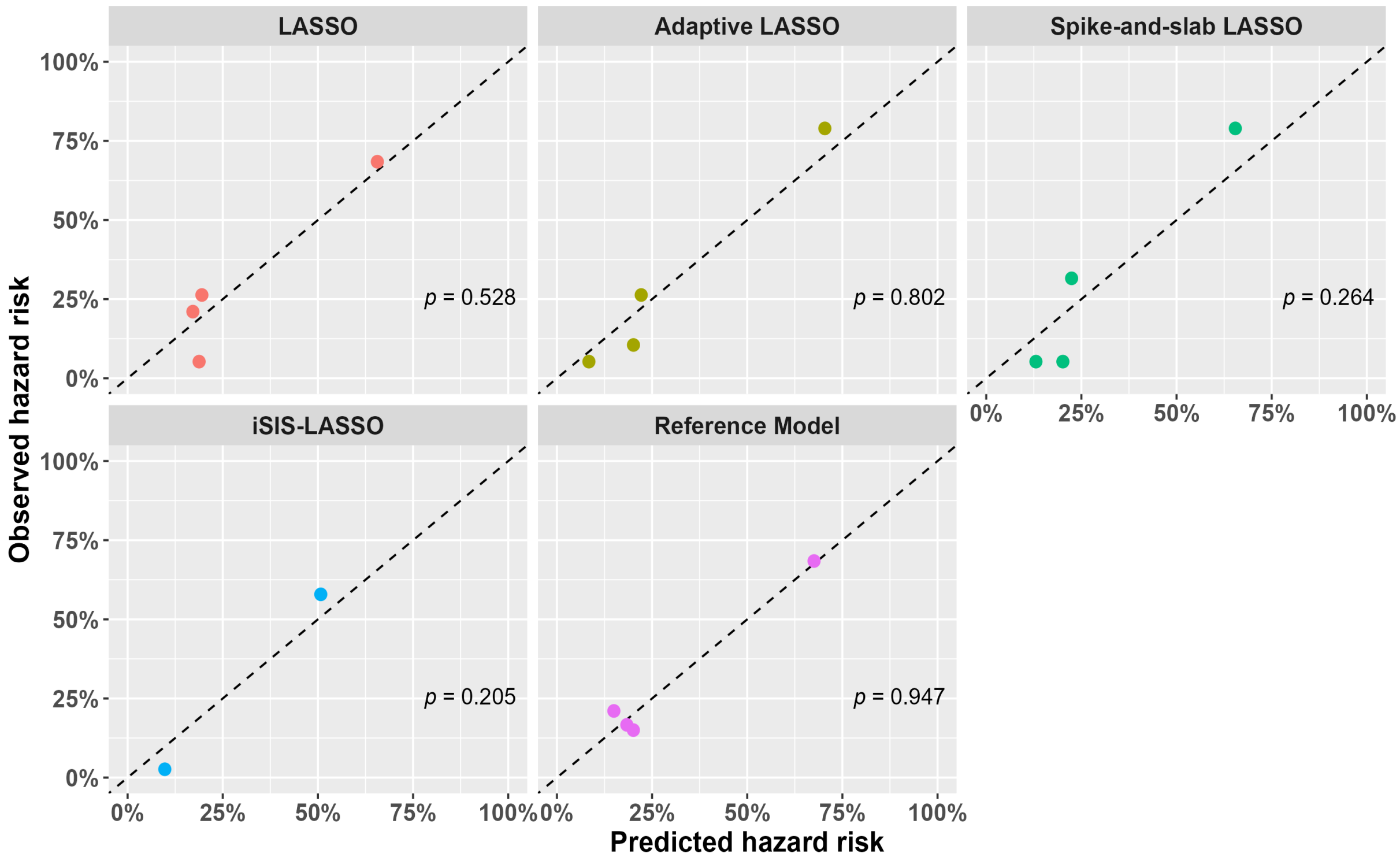

Figure S2.2. Calibration of final prognostic models (with additional adjustment for tumor site) fit on all study participants. Each panel corresponds to a modeling approach (LASSO, adaptive LASSO, spike-and-slab LASSO, or the clinical reference model) and displays the observed versus predicted hazard risk across patient risk groups. Risk groups were defined by quartiles of the model-specific risk score (linear predictor). For the iSIS-LASSO model, calibration was assessed using a median split rather than quartiles due to limited variability in predicted risk scores. The dashed diagonal line represents perfect calibration. The p-value shown in each panel is from a goodness-of-fit test comparing the predicted and observed risks across the quartiles of predicted risk. A p-value < 0.05 indicates poor calibration.

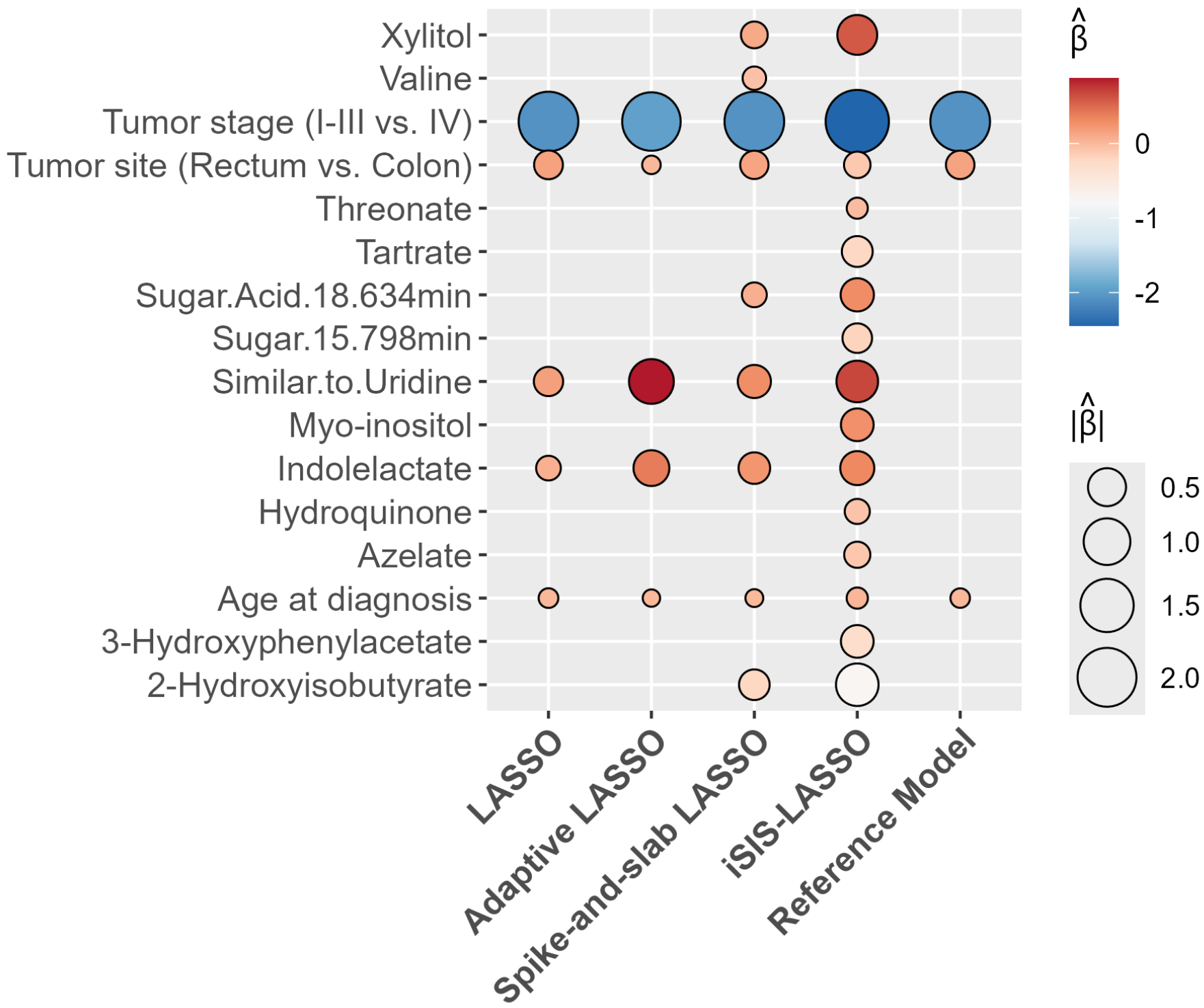

Figure S2.3. Model coefficients of final prognostic models (with additional adjustment for tumor site) fit on all study participants. Each column corresponds to a modeling approach (LASSO, adaptive LASSO, spike-and-slab LASSO, iSIS-LASSO or the clinical reference model), and each row represents a clinical or metabolite feature. Features identified in the corresponding final model are displayed as bubbles. Bubble color represents the sign and magnitude of the estimated log-hazard ratio ($\hat{\beta}$), with red indicating positive associations and blue indicating negative associations. Bubble size is proportional to the absolute value of the estimated log-hazard ratio ($|\hat{\beta}|$). Across penalized models, the core set of metabolites was largely preserved after adjustment for tumor site. A uridine-like metabolite and indolelactate were consistently selected in both analyses, across all penalized approaches, with stable effect directions and only modest attenuation of coefficient magnitudes after tumor-site adjustment, indicating limited confounding by tumor site.

Table S2.1. Model coefficients for selected features in final models with additional adjustment for tumor site *

Feature	LASSO	adpative LASSO	Spike-and-slab	iSIS-LASSO	Reference Model
Xylitol	–	–	0.107	0.594	–
Valine	–	–	-0.047	–	–
Tumor stage (I–III vs. IV)	-2.077	-1.961	-2.076	-2.444	-2.071
Tumor site (Rectum vs. Colon)	0.159	0.006	0.145	-0.101	0.150
Threonate	–	–	–	-0.021	–
Tartrate	–	–	–	-0.217	–
Sugar.Acid.18.634min	–	–	0.069	0.299	–
Sugar.15.798min	–	–	–	-0.179	–
Similar.to.Uridine	0.172	0.876	0.296	0.688	–
Myo-inositol	–	–	–	0.279	–
Indolelactate	0.067	0.401	0.241	0.329	–
Hydroquinone	–	–	–	-0.074	–
Azelate	–	–	–	-0.094	–
Age at diagnosis	0.011	0.005	0.005	0.022	0.010
3-Hydroxyphenylacetate	–	–	–	-0.279	–
2-Hydroxyisobutyrate	–	–	-0.211	-0.742	–

* The reference model includes only tumor stage, age at diagnosis and tumor site; penalized models are adjusted for tumor stage, age at diagnosis and tumor site.

Table S2.2. Calibration assessment of the final models (with additional adjustment for tumor site) using observed and expected event rates across predicted risk quartiles.

Method	Quartile 1 (O / E*)	Quartile 2 (O / E*)	Quartile 3 (O / E*)	Quartile 4 (O / E*)
LASSO	0.053 / 0.188	0.211 / 0.171	0.263 / 0.195	0.684 / 0.656
Adpative LASSO	0.053 / 0.084	0.105 / 0.201	0.263 / 0.221	0.789 / 0.704
Spike-and-slab LASSO	0.053 / 0.131	0.053 / 0.201	0.316 / 0.224	0.789 / 0.654
iSIS-LASSO	0.026 / 0.098**		0.579 / 0.508**	
Reference Model	0.150 / 0.201	0.211 / 0.150	0.167 / 0.184	0.684 / 0.676

* O/E denotes observed (O) and expected (E) event rates within predicted risk groups; E was calculated as the mean predicted event probability within each group.

** For LASSO, adpative LASSO, spike-and-slab LASSO, and the reference model, predicted risks were grouped into quartiles. For iSIS-LASSO, predicted risks were grouped using a median split; values are shown centered across the first two and last two columns to reflect the two-group structure.

Supplement 3 - Simulation Study

Simulation steps

We conducted a simulation study to evaluate the performance of metabolomics-based penalized survival models under varying signal strength (three levels), multicollinearity among predictors(two levels), and censoring rates (three levels). Each scenario consists of 200 iterations. This simulation study aims to evaluate the robustness and finite-sample properties of the penalized Cox models under the empirical correlation structure of the metabolomics data. The main components of simulation procedure are summarized below.

(i) **Design matrix.** We fixed the covariate structure by using the empirical design matrix $\mathbf{X}$ from the ColoCare Study, with $n = 76$ patients (rows) and $p = 121$ metabolites (columns). This preserves the real-world correlation structure among metabolites.

(ii) **Construction of true signal sets.** To study the impact of multicollinearity, we predefined two disjoint sets of true signal metabolites:

- a *high-multicollinearity* set consisting of 3 metabolites with an average variance inflation factor (VIF) of 6.43;
- a *low-multicollinearity* set consisting of 3 metabolites with an average VIF of 1.03.

These sets were treated as the true prognostic metabolites in different simulation scenarios.

(iii) **Specification of true coefficients.** For each scenario, nonzero regression coefficients were assigned to the true signals on the log-hazard scale. We considered three target effect sizes $\beta \in \{0.2,\ 0.4,\ 0.7\}$ to approximate modest, moderate, and strong effects:

- $\exp(0.2) \approx 1.22$ (about a 20% increase in hazard)
- $\exp(0.4) \approx 1.49$ (about a 50% increase in hazard)
- $\exp(0.7) \approx 2.01$ (about a 100% increase in hazard)

For a given target level β, each true nonzero coefficient β_j was drawn independently from a uniform distribution

$$\beta_j \sim \mathrm{Unif}\big(0.8\,\beta,\ 1.2\,\beta\big),$$

introducing mild variation in each iteration for effect sizes within each scenario. The sign of each nonzero coefficient was assigned to be consistent with the observed correlation structure among the signal metabolites (i.e., positively correlated signals were given the same sign, whereas negatively correlated signals were assigned opposite signs). All remaining metabolites were assigned true coefficients of zero.

(iv) **Generation of survival outcomes.** Given the design matrix $\mathbf{X}$ and the specified true coefficient vector, we generated right-censored survival data under three censoring levels: 30%, 50%, and 70%. Each combination of signal set (high *vs.* low multicollinearity), effect size, and censoring rate defined a distinct simulation scenario.

(1) Control of simulated median survival time (target 6 years)

We generated event times from a parametric proportional hazards model with a Weibull baseline. For subject i with covariate vector $\mathbf{X}_i$, the hazard function was

$$h(t \mid \mathbf{X}_i) = h_0(t)\ \exp\big(\mathbf{X}_i^\top \boldsymbol{\beta}\big),$$

where the baseline hazard followed a Weibull form

$$h_0(t) = \lambda \, \gamma \, t^{\gamma - 1},$$

and the corresponding survival function is

$$S(t \mid \mathbf{X}_i) = \exp\bigl\{-\lambda \, t^{\gamma} \exp(\mathbf{X}_i^{\top} \boldsymbol{\beta})\bigr\},$$

with shape parameter fixed at $\gamma = 1.5$, reflecting increase of hazard (risk) over time.

Event times were drawn using inverse transform sampling. Let $U_i \sim \mathrm{Unif}(0, 1)$, then

$$T_i = \left(-\frac{\log U_i}{\lambda \exp(\mathbf{X}_i^{\top} \boldsymbol{\beta})}\right)^{1/\gamma}.$$

To control the median survival time (reflecting the observed data: median survival time is 6 years), we chose the Weibull scale parameter λ so that the median survival equals a prespecified value m_i for each subject. Since

$$S(m_i \mid \mathbf{X}_i) = \exp\bigl\{-\lambda \, m_i^{\gamma} \exp(\mathbf{X}_i^{\top} \boldsymbol{\beta})\bigr\},$$

setting $S(m_i \mid \mathbf{X}_i) = 0.5$ implies

$$\lambda = \frac{\log 2}{m_i^{\gamma} \exp(\mathbf{X}_i^{\top} \boldsymbol{\beta})}.$$

In our simulations we fixed $m_i \equiv 6$ years for all subjects, so that the generated survival

times had a target median of 6 years across scenarios while preserving relative risk contrasts through $\boldsymbol{\beta}$.

(2) Control of censoring rate

Independent time to event T_i and right-censoring times C_i were generated from a parametric distribution governed by a scalar tuning parameter θ (e.g., an exponential distribution with rate θ). Let the observed time and event indicator were

$$Y_i = \min(T_i, C_i), \qquad \Delta_i = \mathbf{1}\{T_i \le C_i\}.$$

Our goal was to choose θ to achieve a target censoring proportion $p^* \in \{0.3, 0.5, 0.7\}$. For a given value of θ, the empirical censoring rate is

$$\hat{p}(\theta) = \frac{1}{n}\sum_{i=1}^{n} \mathbf{1}\{C_i < T_i\} = \frac{1}{n}\sum_{i=1}^{n}(1 - \Delta_i).$$

We then searched for

$$\theta^* = \arg\min_{\theta > 0} \left|\hat{p}(\theta) - p^*\right|$$

using a simple binary search, exploiting the fact that $\hat{p}(\theta)$ increases monotonically with θ:

(a) Choose bounds $0 < \theta_L < \theta_H$ such that $\hat{p}(\theta_L) \le p^* \le \hat{p}(\theta_H)$.

(b) At each iteration, set $\theta_M = (\theta_L + \theta_H)/2$, regenerate censoring times C_i and recompute $\hat{p}(\theta_M)$.

(c) If $\hat{p}(\theta_M) > p^*$ (too much censoring), update $\theta_H \leftarrow \theta_M$; otherwise update $\theta_L \leftarrow \theta_M$.

(d) Stop when $\left|\hat{p}(\theta_M) - p^*\right| \le \varepsilon$ for a small tolerance ε (e.g. $\varepsilon = 0.01$), and take $\theta^* \approx \theta_M$.

This procedure yielded simulated datasets with the desired censoring proportions (30%, 50%, and 70%) while keeping the event-time distribution fixed across censoring scenarios.

(v) **Methods compared.** Across all scenarios, we applied the following methods with consistent model specification described in the method section of the main body of the manuscript:

- LASSO;
- adpative LASSO;
- spike-and-slab LASSO;
- iSIS-LASSO.

(vi) **Performance metrics.** For each method and scenario, we evaluated:

- *Prediction performance:* Harrell's C-index, time-dependent AUC at $1^{st}, 2^{nd}, 3^{rd}, 4^{th}$, and 5th year, and integrated AUC (iAUC) across all follow up time;
- *Feature selection accuracy:* recovery of the full true set (all true signals selected and no noise variables), F_1 score and recall (or true positive rate).
- *Coefficient estimation error:* the ℓ_2-Norm of the difference between estimated and true coefficients, evaluated both on the full coefficient vector and restricted to the true support.

Simulated data quality check

To confirm that the simulated data reflected the prespecified simulation design, we performed several data quality checks across all scenarios. First, the absolute values of the six true log-hazard coefficients clustered tightly around their target magnitudes (0.2, 0.4, and 0.7) under both high- and low-multicollinearity settings, confirming that the signal strength was well controlled (Figure S3.1). Second, Kaplan–Meier estimates of the median survival time were centered around the target of 6 years for the 30% and 50% censoring scenarios, with greater dispersion only under 70% censoring, as expected when events are reduced (Figure S3.2). Third, the realized censoring proportions closely matched the prespecified levels (30%, 50%, 70%) across all effect sizes and correlation structures (Figure S3.3). Together, these checks indicate that the simulated datasets faithfully reflect the planned effect size, median survival time, and censoring rate.

Predictive model performance in the simulation study

In terms of model discrimination, the oracle model (fit with knowledge of the true signals) consistently achieved the highest C-index, iAUC and tAUC across effect sizes and censoring levels, as expected (Figure S3.4, S3.5, S3.6). For all methods, discrimination improved with increasing effect size and declined modestly as censoring increased, with broadly similar patterns under high and low multicollinearity. Across the four penalized approaches, differences in predictive performance were generally small in magnitude. Under low censoring (30%), spike-and-slab LASSO showed the highest discrimination, whereas under heavier censoring ($\geq 50\%$) with moderate-to-strong signals ($\beta > 0.4$), iSIS-LASSO most often exhibited the highest discrimination. When discrimination was examined at specific follow-up horizons, tAUC increased with follow-up time, peaked around 3 or 4 years, and then declined, with heavier censoring

modestly reducing performance. At 2–4 years, spike-and-slab LASSO tended to yield the highest tAUCs under 30% censoring, whereas iSIS-LASSO most often achieved the highest tAUCs when censoring exceeded 50%.

Feature-selection accuracy showed clearer separation between methods (Figure S3.7, S3.8, S3.9). Overall, the F_1 scores increased with larger effect size, lower multicollinearity and lower censoring, but remained far from perfect even in favorable settings. The adpative LASSO model consistently outperformed all other penalized models when censoring rate is ≤ 0.5, while the spike-and-slab LASSO performed the best when censoring rate is high (0.7) and under low multicollinearity. The iSIS-LASSO yielded the lowest F_1 scores, while achieved the best recall (also known as true positive rate or sensitivity) across most scenarios. Full-set recovery was essentially zero for all methods at the smallest effect size ($\beta = 0.2$), but increased at $\beta = 0.4$ and $\beta = 0.7$, particularly with low censoring and low multicollinearity. In these stronger-signal settings, iSIS-LASSO had the largest probability of recovering the complete true set, whereas LASSO, adpative LASSO and spike-and-slab LASSO showed more modest recovery rates, which deteriorated as censoring increased.

Penalized methods showed comparable l_2-Norm errors, with larger variability for procedures that rely on aggressive iterative screening (e.g., iSIS-LASSO) when signals were weak or censoring was heavy (Figure S3.10, S3.11).

While the penalized methods achieve similar discrimination, iSIS-LASSO provides the most reliable recovery of the true signals, at the cost of more variability in coefficient estimation and more false discovery rate, especially under challenging high-censoring or highly collinear settings.

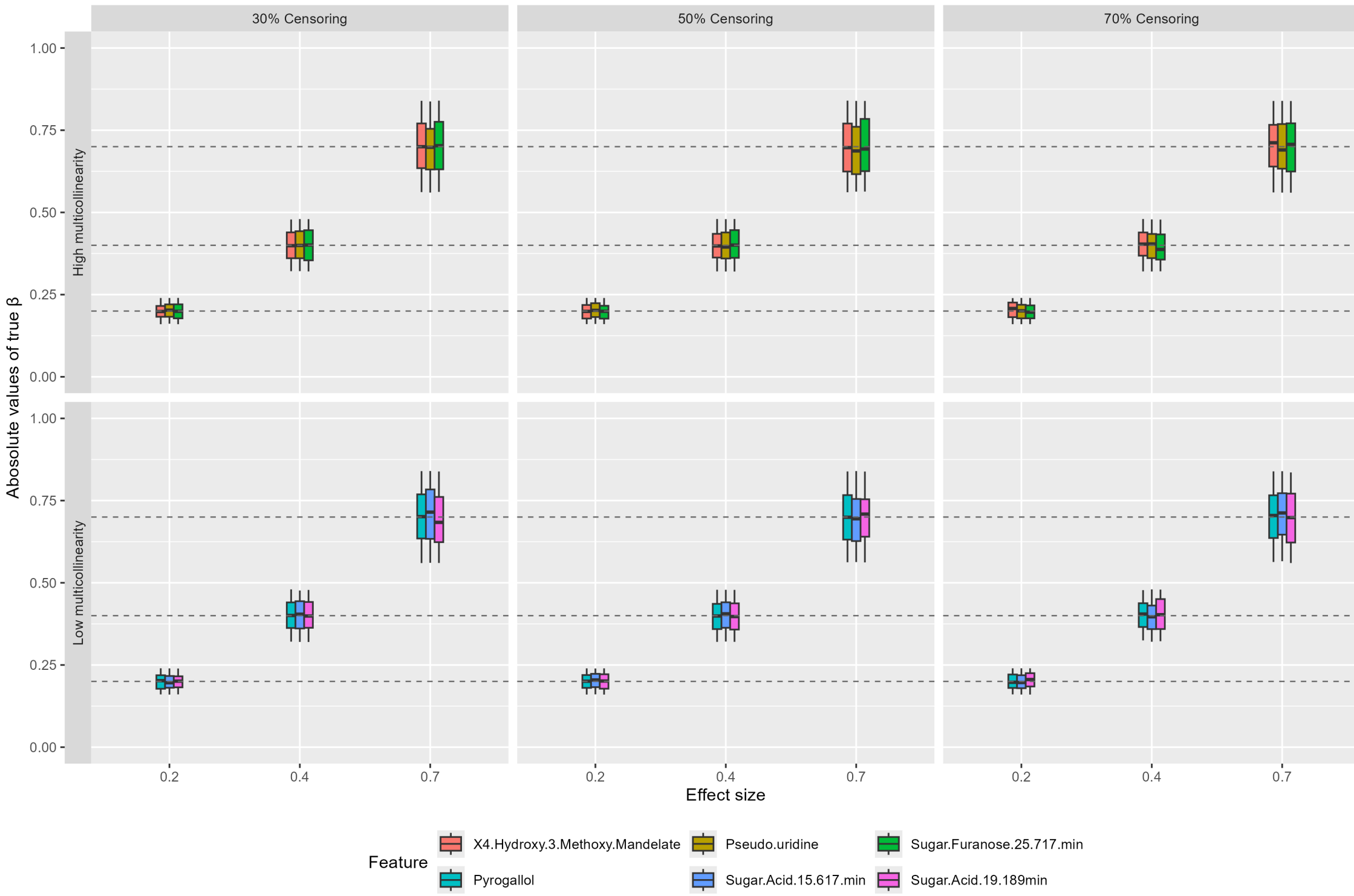

Figure S3.1. Distribution of simulated true coefficients across simulation scenarios. Boxplots of the absolute true log-hazard coefficients ($|\beta|$) used in the simulations by target effect size (0.2, 0.4, and 0.7; x-axis), correlation structure (high vs. low multicollinearity; rows), and censoring level (30%, 50%, and 70%; columns). Each boxplot summarizes 200 simulated draws for the three active metabolites within each correlation structure. Colors denote individual metabolites, and horizontal dashed lines indicate the target effect sizes, demonstrating that the simulated coefficients were centered on the intended values across all scenarios.

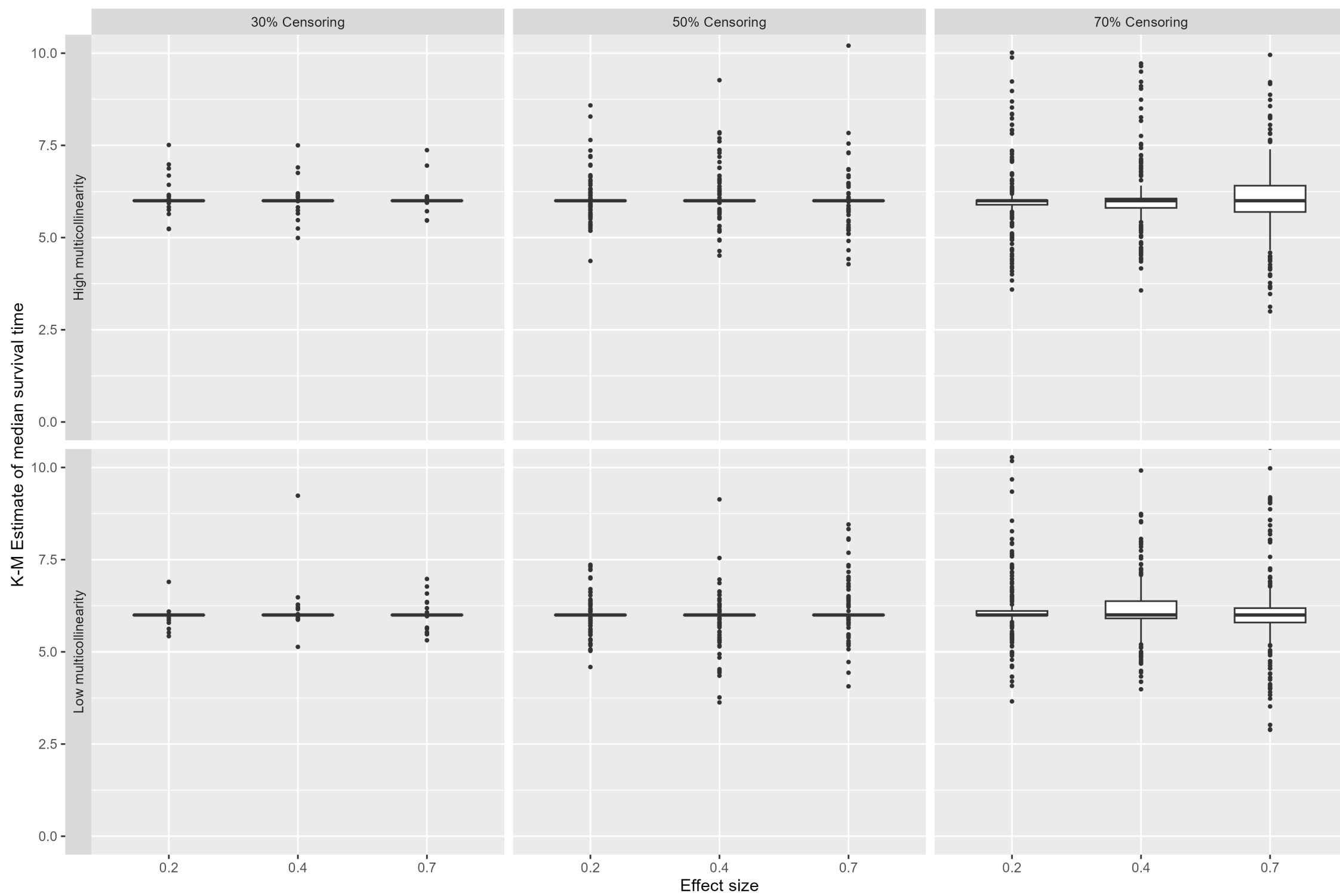

Figure S3.2. Kaplan–Meier estimates of the median survival time across simulation settings. Each box shows the distribution of median survival time across simulated survival data over 200 replicates by target effect size (0.2, 0.4, and 0.7; x-axis), correlation structure (high vs. low multicollinearity; rows), and censoring level (30%, 50%, and 70%; columns). Across all scenarios, the empirical medians cluster around the target value of 6 years, indicating successful control of the underlying Weibull baseline; variability increases with higher censoring, especially under 70% censoring, but without systematic drift away from the intended median.

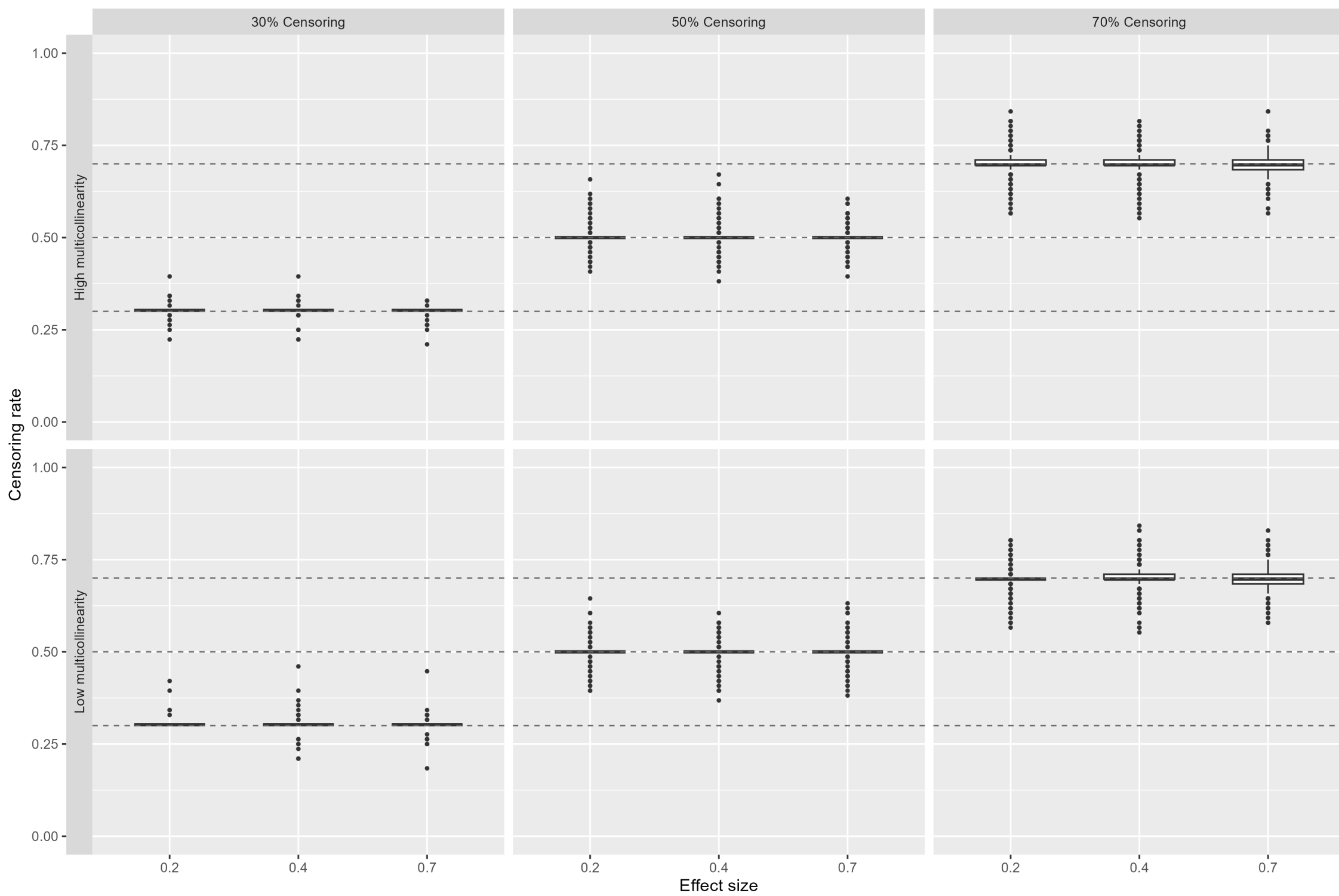

Figure S3.3. Empirical censoring rates across simulation scenarios. Each panel shows the distribution of observed censoring proportions over 200 simulated datasets by target effect size (0.2, 0.4, and 0.7; x-axis), correlation structure (high vs. low multicollinearity; rows), and censoring level (30%, 50%, and 70%; columns). Horizontal dashed lines indicate the target censoring rates. Across all settings, the realized censoring proportions cluster closely around their targets with only modest variability, confirming that the binary–search achieved the desired censoring levels regardless of effect size or correlation structure.

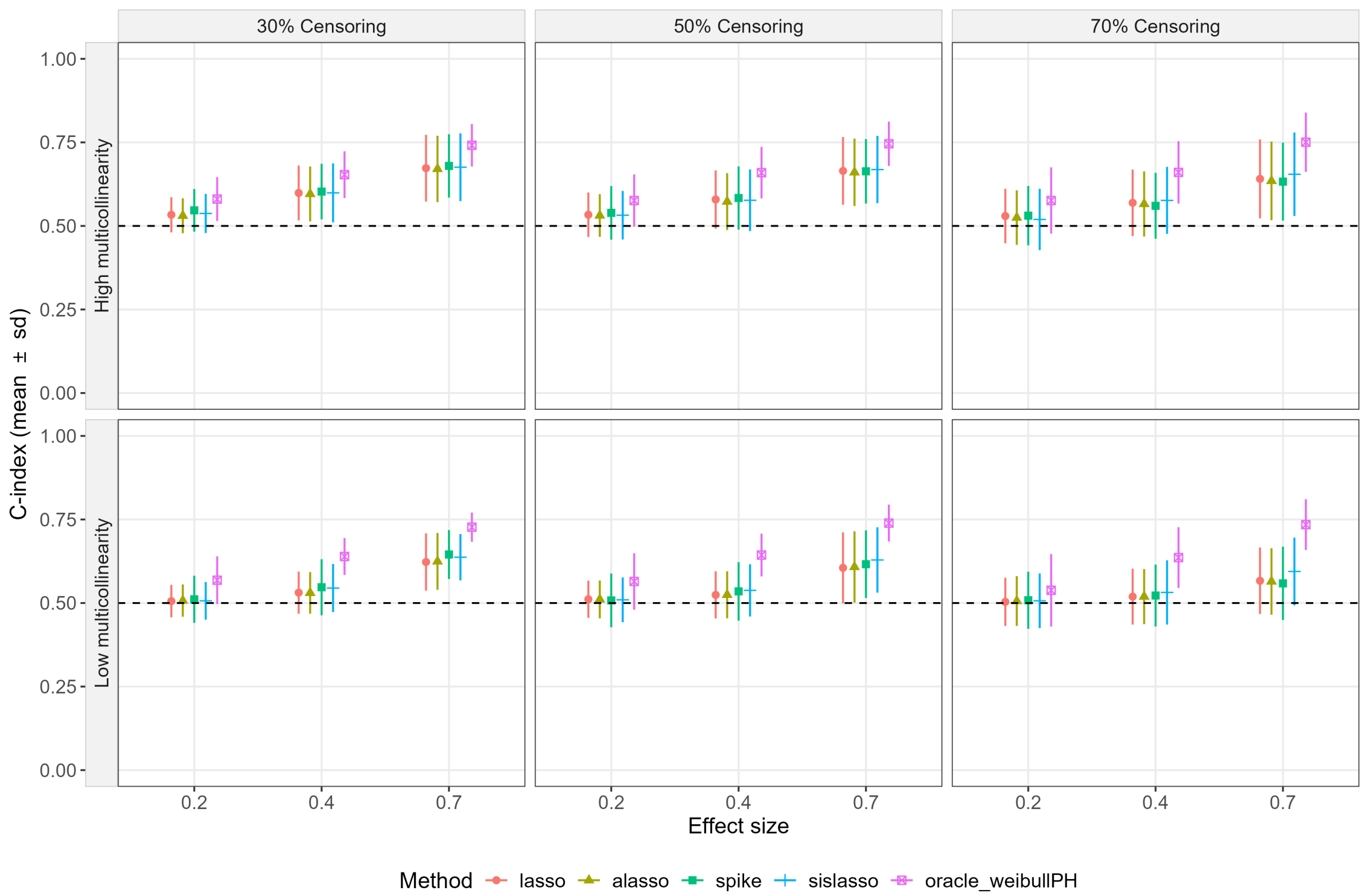

Figure S3.4. Comparison of C-index across methods and simulation scenarios. Mean C-indices (points) and standard deviations (bars) across 200 simulation replicates for each method across effect sizes (0.2, 0.4, 0.7; x-axis), censoring levels (30%, 50%, 70%; columns), and correlation structures (high *vs.* low multicollinearity; rows). The dashed line indicates no better than random prediction (C-index = 0.5). Discrimination increased with larger effect sizes and was only modestly attenuated by heavier censoring. The oracle Weibull proportional hazards model (fit using the true active set) achieved the highest C-indices across all settings, representing an upper bound on achievable performance. Among the penalized methods, performance was broadly similar, with Spike-and-slab LASSO performing best at low censoring (30%) and iSIS-LASSO achieving the highest C-indices under higher censoring ($> 50\%$) and larger effect sizes (> 0.4).

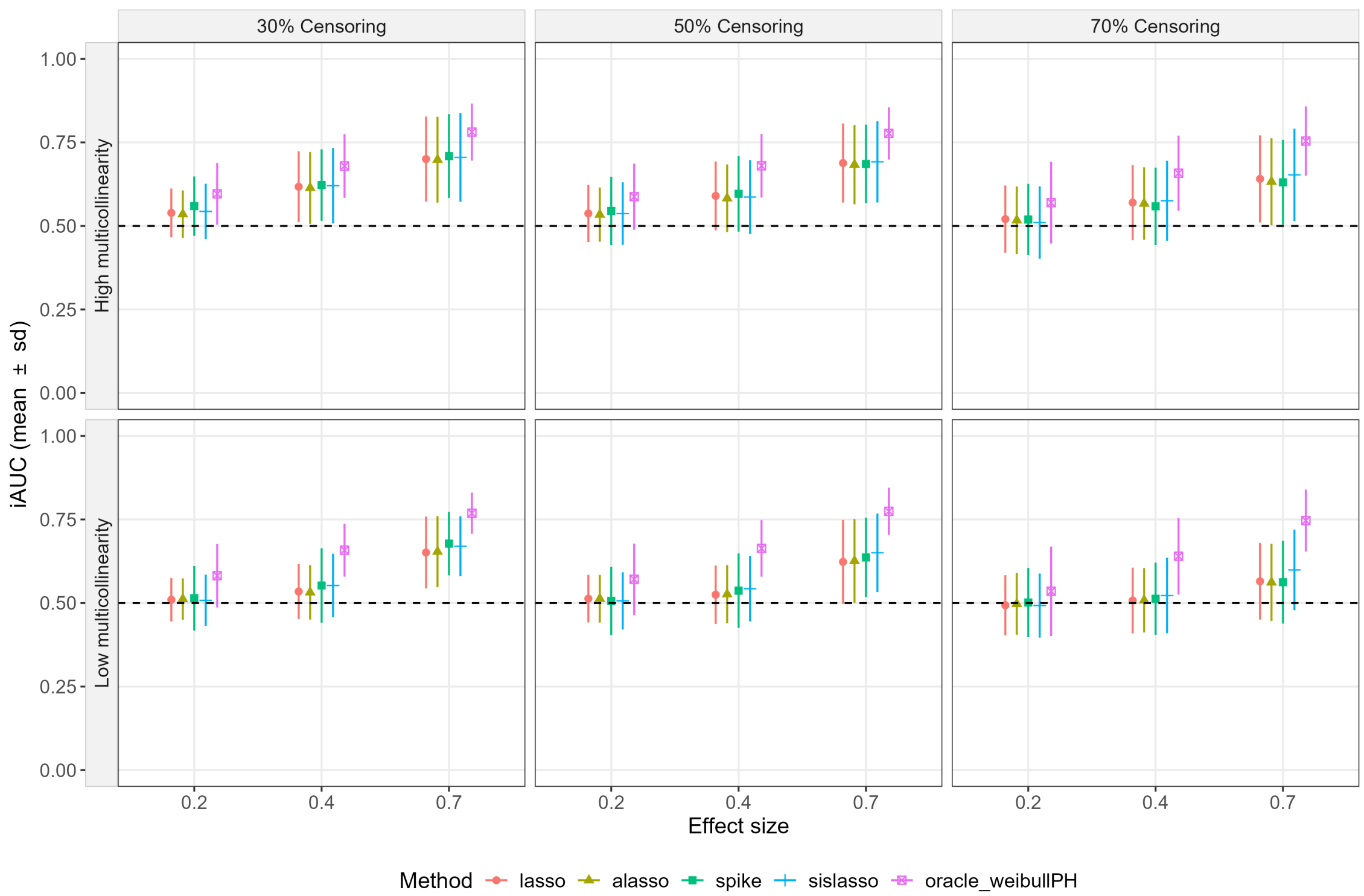

Figure S3.5. Comparison of iAUC across methods and simulation scenarios. Mean iAUCs (points) and standard deviations (bars) across 200 simulation replicates for each method across effect sizes (0.2, 0.4, 0.7), censoring levels (30%, 50%, 70%; columns), and correlation structures (high *vs.* low multicollinearity; rows). The dashed line indicates no better than random prediction (iAUC = 0.5). Discrimination increased with larger effect sizes and was only modestly attenuated by heavier censoring. The oracle Weibull proportional hazards model (fit using the true active set) achieved the highest iAUCs across all settings, representing an upper bound on achievable performance. Among the penalized methods, performance was broadly similar, with Spike-and-slab LASSO performing best at low censoring (30%) and iSIS-LASSO achieving the highest iAUCs under higher censoring ($> 50\%$) and larger effect sizes (> 0.4).

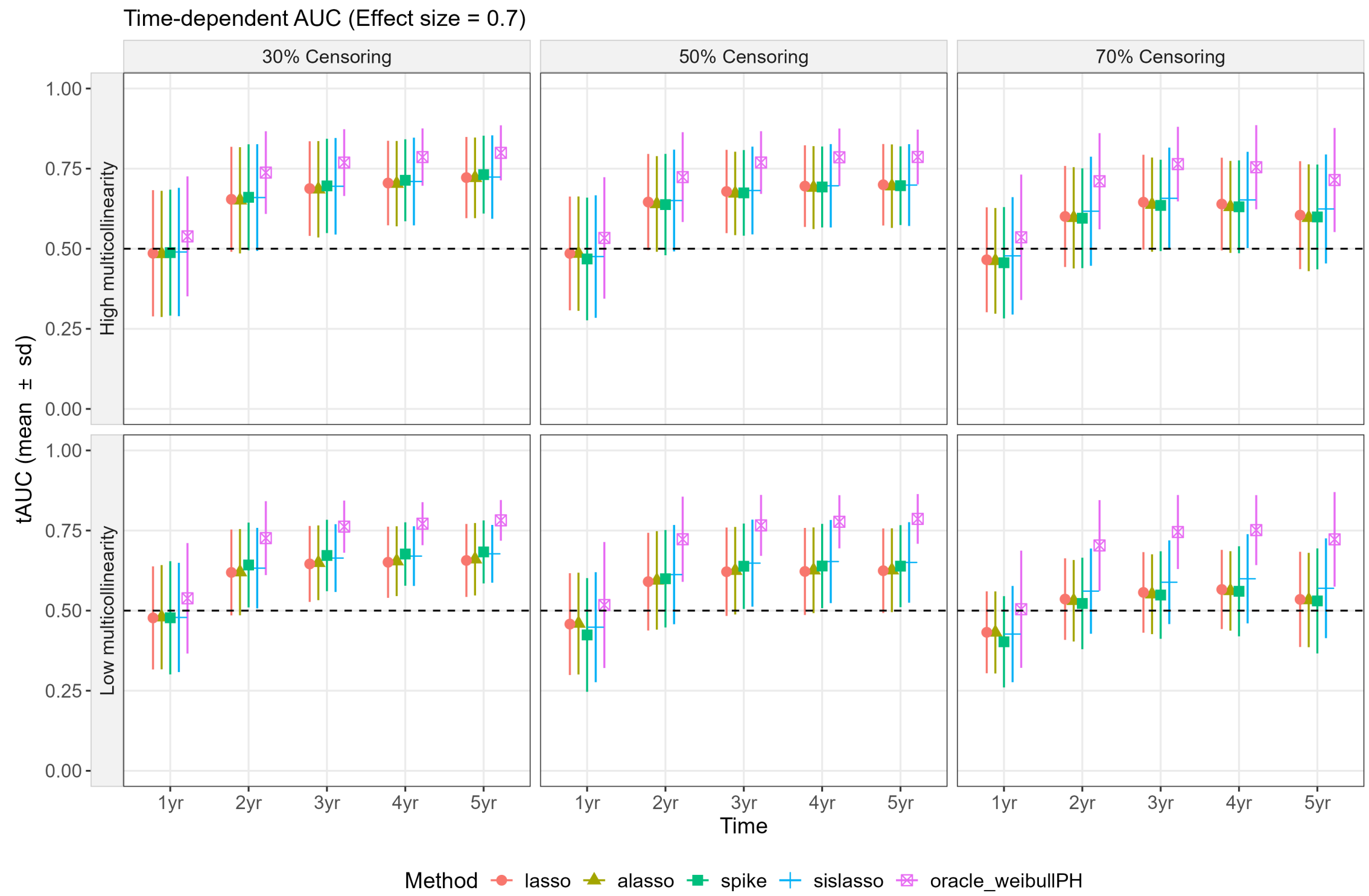

Figure S3.6. Comparison of time-dependent AUCs across methods and simulation scenarios. Mean time-dependent AUCs (tAUCs; points) and standard deviations (error bars) across 200 simulation replicates for each method at effect size 0.7 by censoring levels (30%, 50%, 70%; columns) and correlation structures (high *vs.* low multicollinearity; rows), evaluated at 1–5 years. The dashed line indicates random discrimination (tAUC $= 0.5$). Discrimination was limited at 1 year but became evident after 2 years (consistent with results for $\beta < 0.7$). Performance increased with follow-up time, peaked around years 3-4, and declined thereafter, with heavier censoring modestly reducing performance. Among the penalized methods, spike-and-slab LASSO performed best under 30% censoring, whereas iSIS-LASSO achieved the highest tAUCs under higher censoring ($\geq$50%). The oracle Weibull proportional hazards model (fit using the true active set) consistently achieved the highest tAUCs, representing the upper bound on performance.

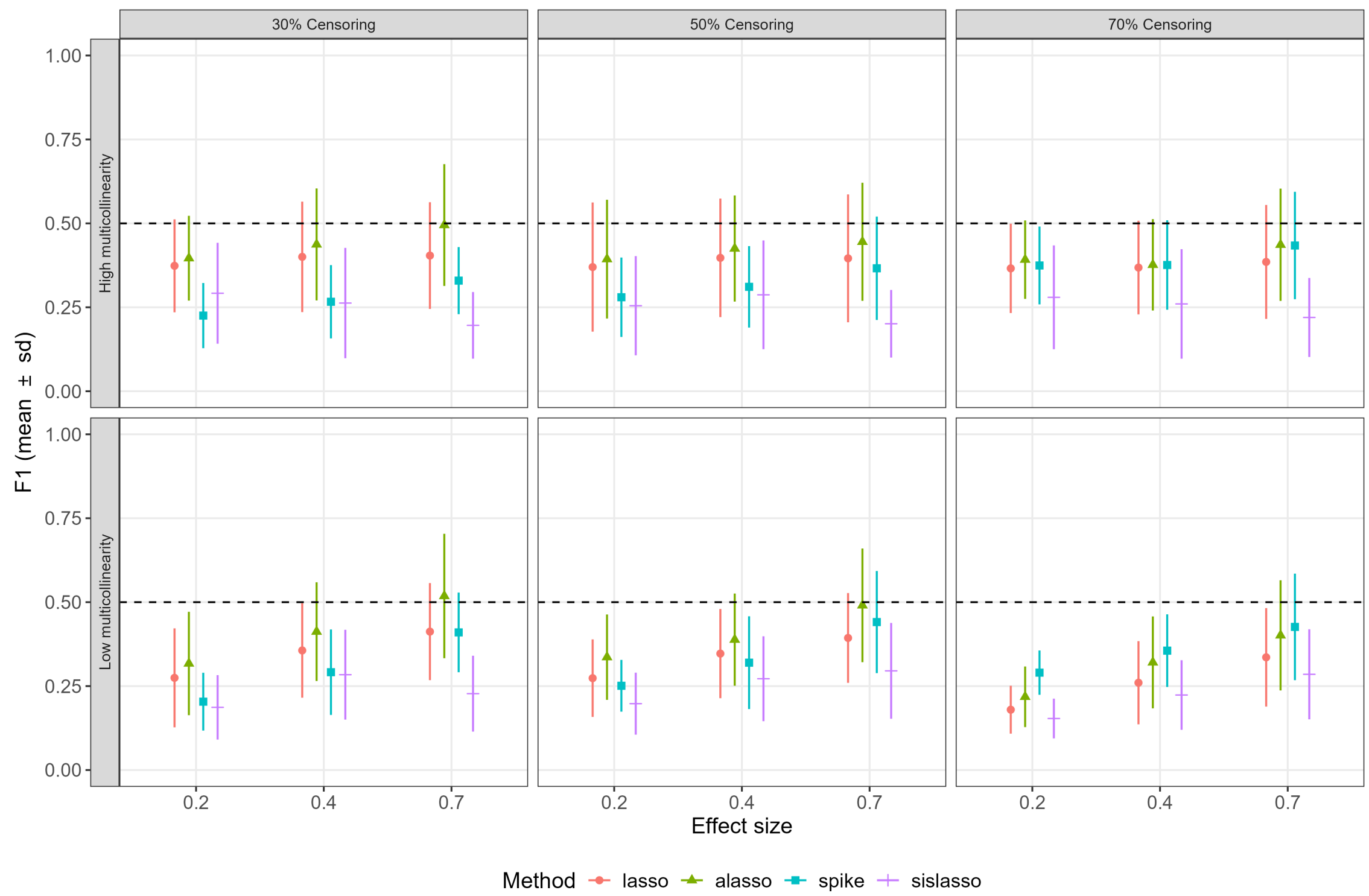

Figure S3.7. Comparison of feature selection F_1-score across methods and simulation scenarios. Mean F_1-scores (points) and standard deviations (error bars) across 200 simulation replicates for recovery of the true signal set by method, effect sizes (0.2, 0.4, 0.7), censoring levels (30%, 50%, 70%; columns), and correlation structures (high *vs.* low multicollinearity; rows). F_1-scores increase with larger effect size and were generally higher under lower censoring and lower multicollinearity. Adpative LASSO consistently achieved the highest F_1-scores, whereas iSIS-LASSO yielded had the lowest F_1-scores across most scenarios, likely reflecting its higher false discovery rate.

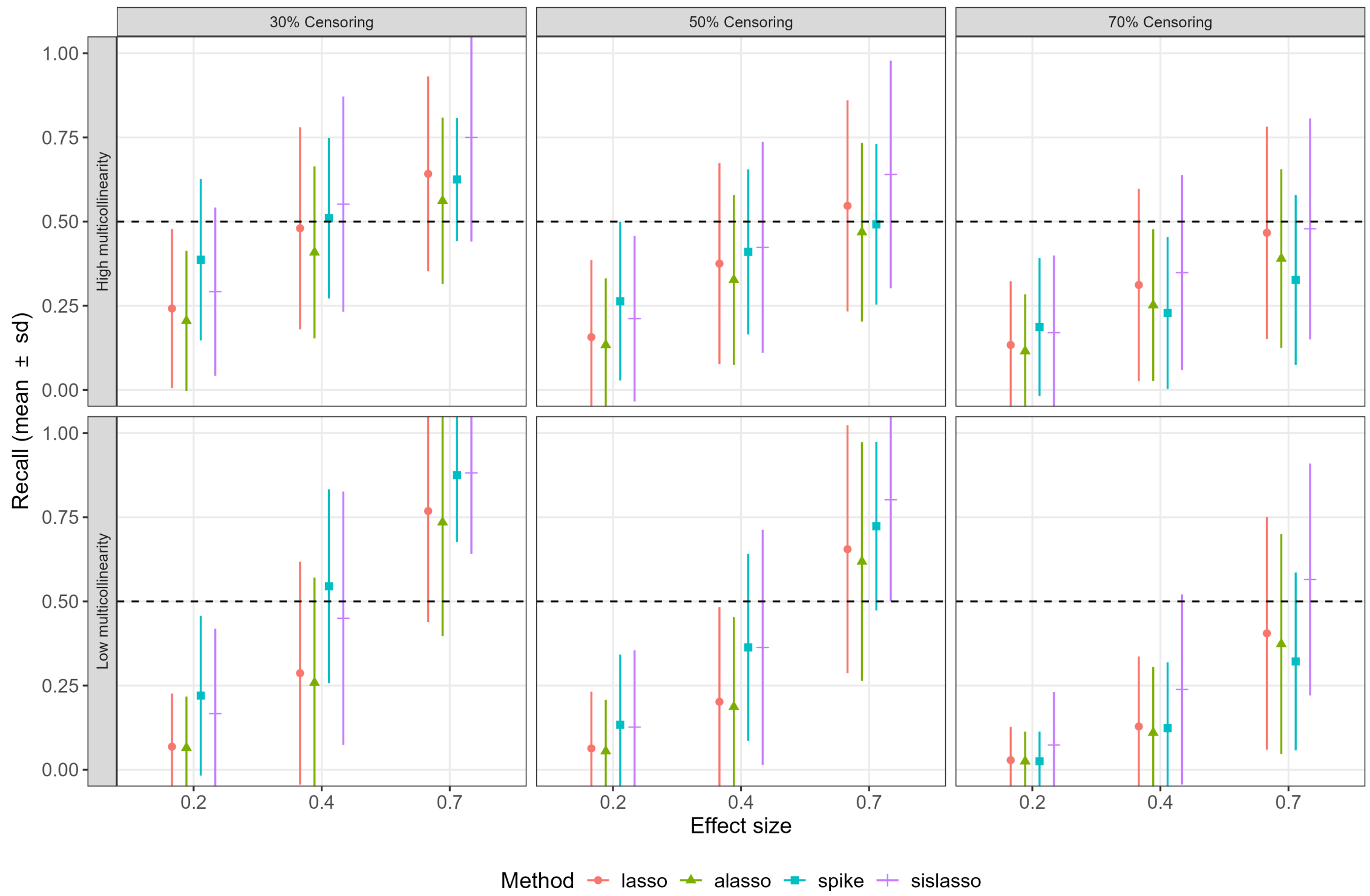

Figure S3.8. Comparison of recall across methods and simulation scenarios. Mean recall (points) and standard deviations (error bars) across 200 simulation replicates for each method by effect sizes (0.2, 0.4, 0.7), censoring levels (30%, 50%, 70%; columns), and correlation structures (high *vs.* low multicollinearity; rows). The dashed line indicates a reference recall of 0.5. Recall increased with larger effect sizes and decreased with heavier censoring, particularly under high multicollinearity. Spike-and-slab LASSO achieved the highest recall under low censoring (30%), particularly with low multicollinearity, but its advantage diminished as censoring increased, whereas iSIS-LASSO consistently achieved the highest recall when $\beta \geq 0.4$ and censoring $\geq 50\%$.

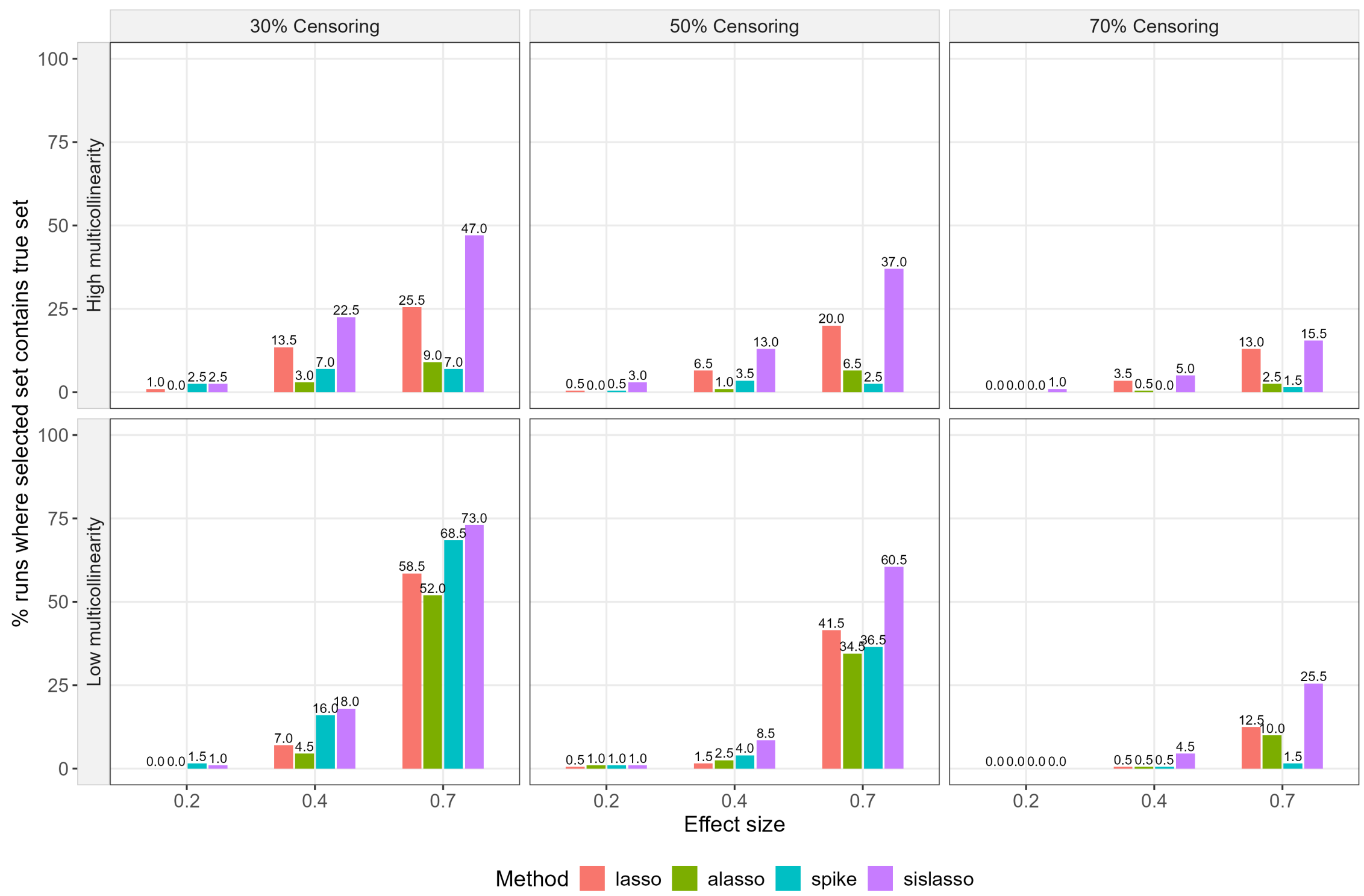

Figure S3.9. Comparison of full-set recovery across methods and simulation scenarios. Bars show the percentage of 200 simulation replicates in which all three true signals were correctly selected ("full-set recovery") for each method by effect sizes (0.2, 0.4, 0.7), censoring levels (30%, 50%, 70%; columns), and correlation structures (high *vs.* low multicollinearity; rows). Full-set recovery was negligible at the smallest effect size ($\beta = 0.2$) and increased with larger effect sizes, particularly under lower censoring and low multicollinearity. Across scenarios, iSIS–LASSO most frequently achieves full-set recovery, especially at ($\beta = 0.7$), whereas recovery declined for all methods with heavier censoring.

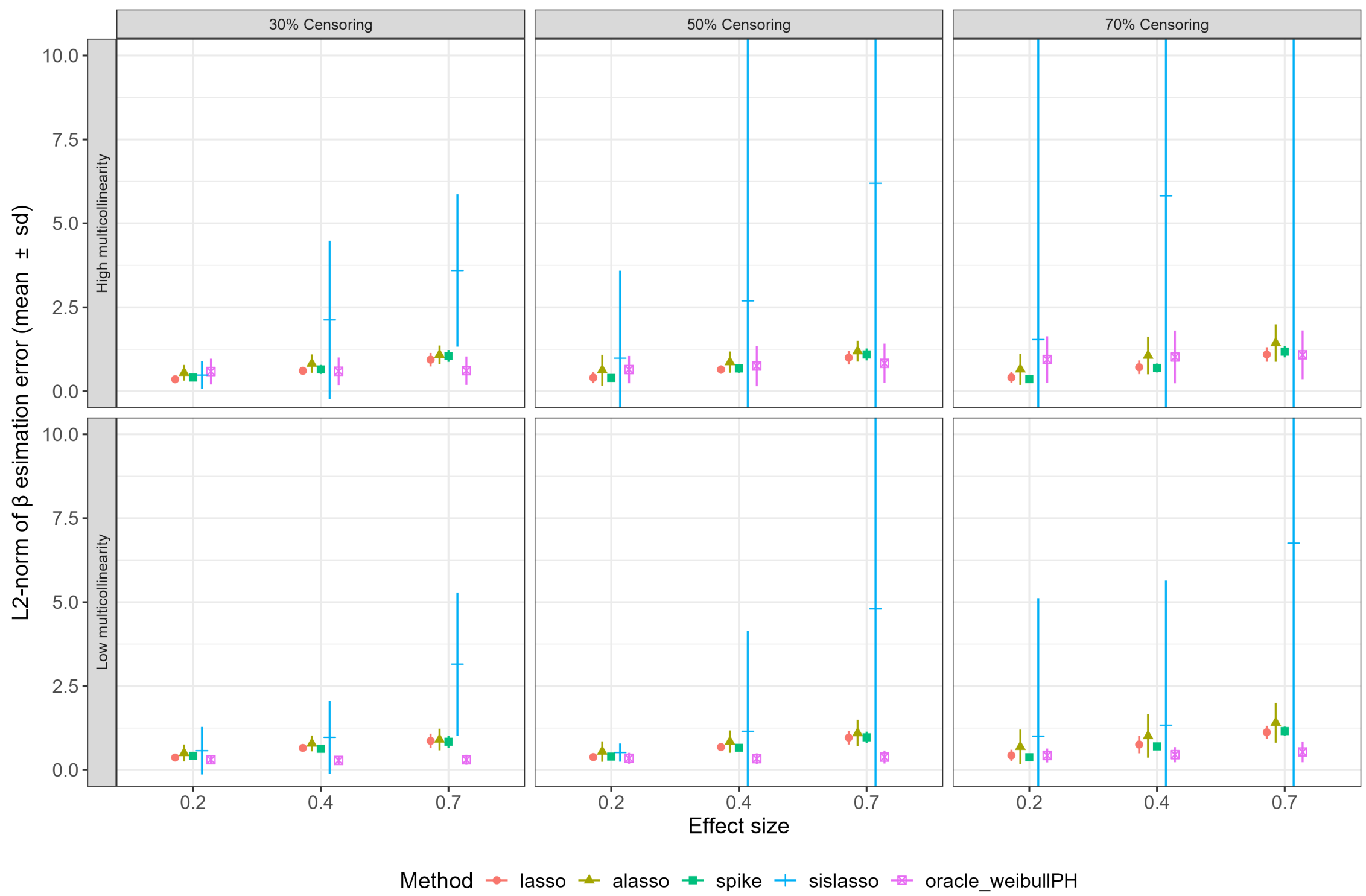

Figure S3.10. Comparison of ℓ_2-Norm of coefficient estimation error on the full support across methods and simulation scenarios. Mean ℓ_2-Norm of the coefficient estimation error ($\left\|\hat{\beta}_{\mathbf{full}} - \beta_{\mathbf{full}}\right\|_2$; points) and standard deviations (error bars) crosss 200 simulation replicates for each method by effect sizes (0.2, 0.4, 0.7), censoring levels (30%, 50%, 70%; columns), and correlation structures (high *vs.* low multicollinearity; rows). The oracle Weibull proportional hazards model (fit using the true active set) achieved the smallest estimation error across all scenarios. Among the penalized methods, LASSO, adaptive LASSO, and spike-and-slab LASSO showed similar estimation errors that increased with heavier censoring. In contrast, iSIS-LASSO exhibited substantially greater estimation error and variability, particularly under high multicollinearity, likely reflecting its higher false discovery rate.

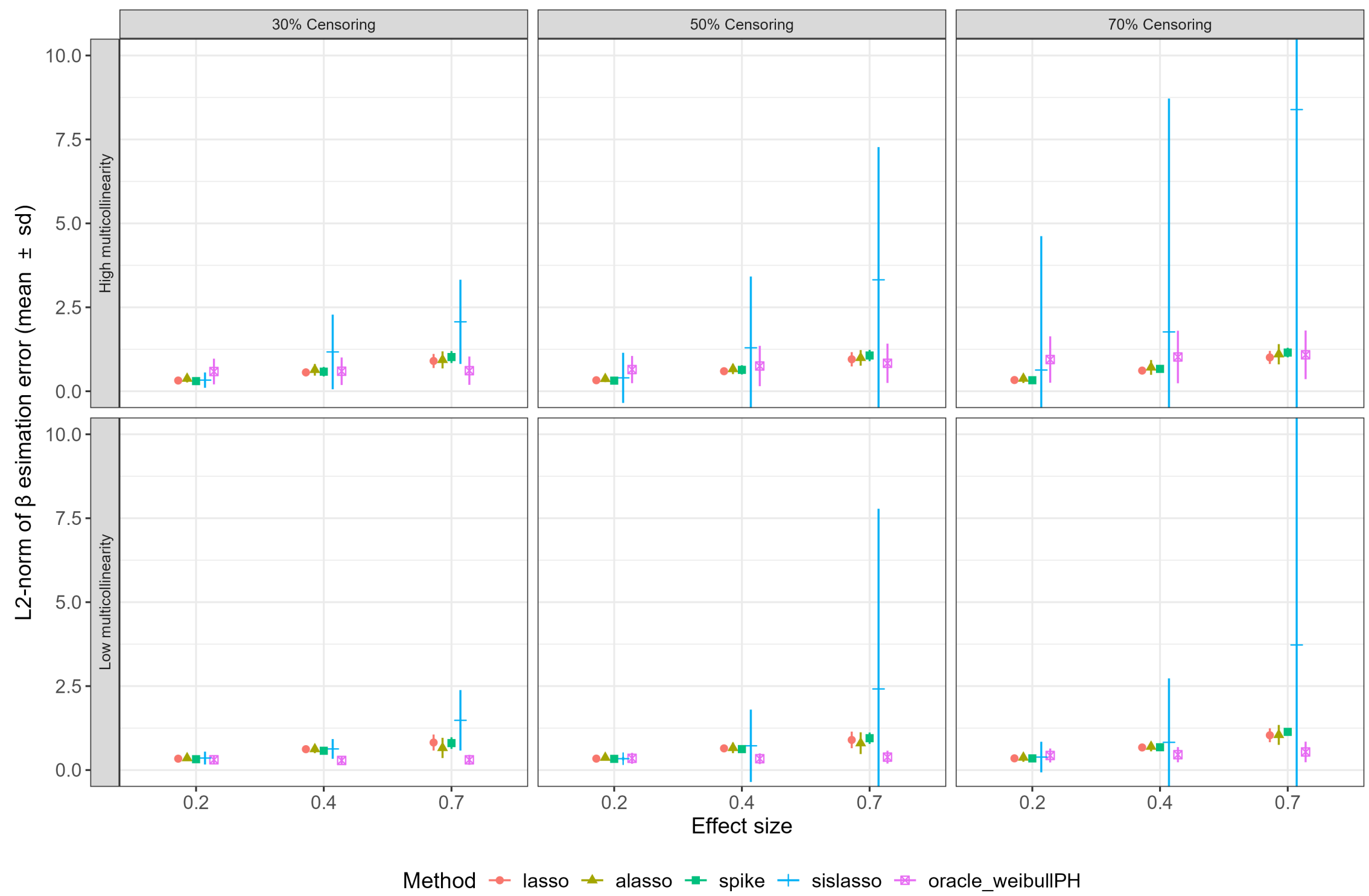

Figure S3.11. Comparison of ℓ_2-Norm of coefficient estimation error on the true support across methods and simulation scenarios. Mean ℓ_2-Norm of the coefficient estimation error on full support ($\left\|\hat{\boldsymbol{\beta}}_{\mathbf{true}} - \boldsymbol{\beta}_{\mathbf{true}}\right\|_{\mathbf{2}}$; points) and standard deviations (error bars) crosss 200 simulation replicates for each method by effect sizes (0.2, 0.4, 0.7), censoring levels (30%, 50%, 70%; columns), and correlation structures (high *vs.* low multicollinearity; rows). Estimation errors were smaller than those for the full-support metric. The oracle Weibull proportional hazards model (fit using the true active set) consistently achieved the smallest estimation error. Among the penalized methods, LASSO, adaptive LASSO, and spike-and-slab LASSO showed similar estimation errors across scenarios, whereas iSIS-LASSO exhibited substantially greater estimation error and variability, particularly under high multicollinearity.

List of Figures

Figure 1 Model discrimination assessment

Figure 2 Model calibration assessment

Figure 3 Model coefficients estimation from final models

Figure S1.1 Enriched metabolite sets based on selected metabolites (Models: LASSO, adaptive LASSO, and iSIS-LASSO)

Figure S1.2 Enriched metabolite sets based on selected metabolites (Model: spike-and-slab LASSO)

Figure S2.1 Model discrimination assessment (with additional adjustment for tumor site).

Figure S2.2 Model calibration assessment (with additional adjustment for tumor site)

Figure S2.3 Model coefficient estimation from final models (with additional adjustment for tumor site)

Figure S3.1 Distribution of simulated model coefficients

Figure S3.2 Distribution of median survival time of simulated survival data

Figure S3.3 Distribution of censoring of simulated survival data

Figure S3.4 Model performance in simulation study: C-index

Figure S3.5 Model performance in simulation study: iAUC

Figure S3.6 Model performance in simulation study: tAUC

Figure S3.7 Model performance in simulation study: F_1 Score

Figure S3.8 Model performance in simulation study: Recall of true signals

Figure S3.9 Model performance in simulation study: Proportion of full-set recovery

Figure S3.10 Model performance in simulation study: ℓ_2-Norm of β estimation error, full supports

Figure S3.11 Model performance in simulation study: ℓ_2-Norm of β estimation error, true supports

List of Tables

Table 1 Patient characteristics at baseline

Table 2 Model coefficients for selected features in final models

Table 3 Model calibration assessment

Table S1.1 Model specifications of penalized Cox modeling

Table S1.2 Calibration assessment procedure

Table S1.3 Nested cross-validation estimate of global discrimination performance with bootstrap 95% confidence intervals (1,000 bootstrap repetitions).

Table S1.4 Nested cross-validation estimate of global discrimination improvement over the reference model with bootstrap 95% confidence intervals (1,000 bootstrap repetitions).

Table S1.5 Nested cross-validation estimate of time-dependent AUC (tAUC) with bootstrap 95% confidence intervals (1,000 bootstrap repetitions).

Table S1.6 Nested cross-validation estimate of time-dependent AUC (tAUC) improvement over the reference model with bootstrap 95% confidence intervals (1,000 bootstrap repetitions).

Table S2.1 Model coefficients for selected features in final models with additional adjustment for tumor site

Table S2.2 Model calibration assessment for final models with additional adjustment for tumor site
